# Integrated Excitatory/Inhibitory Imbalance and Transcriptomic Analysis Reveals the Association between Dysregulated Synaptic Genes and Anesthetic-Induced Cognitive Dysfunction

**DOI:** 10.3390/cells11162497

**Published:** 2022-08-11

**Authors:** Yasheng Yan, Sarah Logan, Xiaojie Liu, Bixuan Chen, Congshan Jiang, Thiago Arzua, Ramani Ramchandran, Qing-song Liu, Xiaowen Bai

**Affiliations:** 1 Department of Cell Biology, Neurobiology & Anatomy, Medical College of Wisconsin, Milwaukee, WI 53226, USA; 2 Department of Pharmacology, Medical College of Wisconsin, Milwaukee, WI 53226, USA; 3 Department of Anesthesiology, Medical College of Wisconsin, Milwaukee, WI 53226, USA; 4 Department of Pediatrics, Division of Neonatology, Medical College of Wisconsin, Milwaukee, WI 53226, USA

**Keywords:** propofol, excitatory and inhibitory imbalance, transcriptomic, synaptic genes, cognitive dysfunction

## Abstract

Emerging evidence from human epidemiologic and animal studies has demonstrated that developmental anesthesia neurotoxicity could cause long-term cognitive deficits and behavioral problems. However, the underlying mechanisms remain largely unknown. We conducted an electrophysiological analysis of synapse activity and a transcriptomic assay of 24,881 mRNA expression on hippocampal tissues from postnatal day 60 (P60) mice receiving propofol exposure at postnatal day 7 (P7). We found that developmentally propofol-exposed P60 mouse hippocampal neurons displayed an E/I imbalance, compared with control mice as evidenced by the decreased excitation and increased inhibition. We found that propofol exposure at P7 led to the abnormal expression of 317 mRNAs in the hippocampus of P60 mice, including 23 synapse-related genes. Various bioinformatic analyses revealed that these abnormally expressed synaptic genes were associated with the function and development of synapse activity and plasticity, E/I balance, behavior, and cognitive impairment. Our findings suggest that the altered E/I balance may constitute a mechanism for propofol-induced long-term impaired learning and memory in mice. The transcriptomic and bioinformatic analysis of these dysregulated genes related to synaptic function paves the way for development of therapeutic strategies against anesthetic neurodegeneration through the restoration of E/I balance and the modification of synaptic gene expression.

## 1. Introduction

Each year, up to 2% of all pregnant women and over 1.3 million children (<5 years old) undergo surgical procedures involving general anesthesia in the United States [1,2]. Emerging evidence from animal studies has demonstrated that general anesthetics (GAs) could cause developmental neurotoxicity [3,4,5,6,7,8,9]. Meanwhile, growing numbers of human studies and meta-analyses of prospective human data studies suggest that developing GA exposure is associated with long-lasting neurodevelopmental deficits [10,11,12,13,14,15]. The developing brain during the rapid synaptogenesis period was more vulnerable to anesthetics [16]. Acute anesthetic-induced developmental neurotoxicity (AIDN) was evidenced by pathologic changes in the brain (e.g., brain cell death) and abnormal neuronal development [1,17,18]. The chronic neurotoxic effects of developmental GA exposure include impaired synaptic plasticity and cognitive and behavioral deficits, however, the underlying mechanisms are largely unknown [19].

Identifying factors that contribute to the anesthetic-induced long-term cognitive deficits is an area of active research. Many studies have focused on defining how GA affects the structural integrity of neurons and, in turn, synapse changes, which correlates with cognitive decline. Deficits in synaptic function correlate with cognitive decline and abnormal behaviors in many neurodegenerative diseases [20,21]. Each neuron in the brain can receive tens of thousands of excitatory and inhibitory synaptic inputs. The information transfer in the brain relies on a functional balance between excitatory and inhibitory (E/I) networks. Under normal conditions, the ratio of E/I input remains stable, termed E/I balance. This balance involves the maintenance of appropriate ratios of E/I synaptic inputs [22,23,24]. Changes in the E/I balance have been linked to different brain states. An increased inhibition or a reduced synaptic excitation occurs under anesthesia [25,26]. In many pathological conditions, this fine balance is also perturbed, leading to excessive or diminished excitation relative to inhibition. This is termed E/I imbalance and is reflected in network dysfunction. E/I imbalance has emerged as an important contributor to the etiology of many neurodevelopmental and neurodegenerative diseases, as well as psychiatric problems [27,28,29,30]. Our hypothesis lies in an imbalance in E/I synaptic networks. It is thus possible that anesthetics could induce E/I imbalance, thereby leading to impaired cognition.

Abnormal expression of synaptic genes has been associated with age- and AD-related cognitive declines [31,32]. However, whether developmental anesthetic exposure induces long-term changes of synaptic genes is not known. Propofol decreases neuronal activity and excitability through the activation of GABA_A_ (γ-aminobutyric acid type A) receptor [33,34]. Propofol was shown to induce similar cognitive dysfunction as observed in other GA-exposed animal models [1,35,36,37]. The current study focused on the investigation on the long-term effect of a brief developmental propofol exposure at postnatal day 7 (P7) on E/I balance in hippocampal CA1 pyramidal neurons in adult (P60) mice. Genomics studies of transcriptomics have become increasingly important tools to understand the molecular basis of synaptopathies. Thus, to further understand the molecular mechanisms of the anesthetic-induced synaptic dysfunction and cognitive deficits, we conducted the transcriptomic analysis of adult mouse hippocampi and performed in-depth bioinformatics analysis of the relationship that synaptic genes had with the anesthetic-induced chronic neurodegeneration.

## 2. Materials and Methods

### 2.1. Propofol Exposure

All animal experiments described were approved by the Institutional Animal Care and Use Committee at the Medical College of Wisconsin. C57BL/6 mice (The Jackson Laboratory, Bar Harbor, ME, USA) received a propofol dose on P7 when the developing brain is vulnerable to anesthetics, also the equivalent of third-trimester embryo in humans [38]. Both male and female mice were included in the studies and randomly distributed into propofol or control groups. P7 mice were placed in a temperature-controlled incubator (37 °C) and received an intraperitoneal injection of propofol (Zoetis, Parsippany, NJ, USA) or 10% intralipid (Fresenius Kabi, Bad Homburg, Germany) as a vehicle control. During anesthesia, the rectal temperature of pups was maintained at 37 ± 1 °C. In total, either 2 or 4 injections of 50 mg/kg body weight of propofol were administered, with a 90 min interval. This dose of propofol was selected based on previous reports from us and others showing that the dosage of propofol required to induce a surgical plane of anesthesia in mice was 200 mg/kg and sub-anesthetic doses of 50 mg/kg propofol could induce neuroapoptosis [35,38,39,40]. This dose could result in an anesthetic depth with the loss of righting reflex but a remaining response to toe pinch. One injection of propofol maintained anesthesia in mice for ~90 min [39]. Thus, we injected propofol every 90 min for a total of 2 or 4 times to maintain 3 h- or 6 h-anesthesia. The mice were either euthanized for brain tissue harvest following propofol exposure or placed back to home cages used for chronic propofol neurotoxicity studies of cognition, gene expression, and E/I balance on P60 mice.

### 2.2. Immunofluorescence Staining

**Figure 1:**
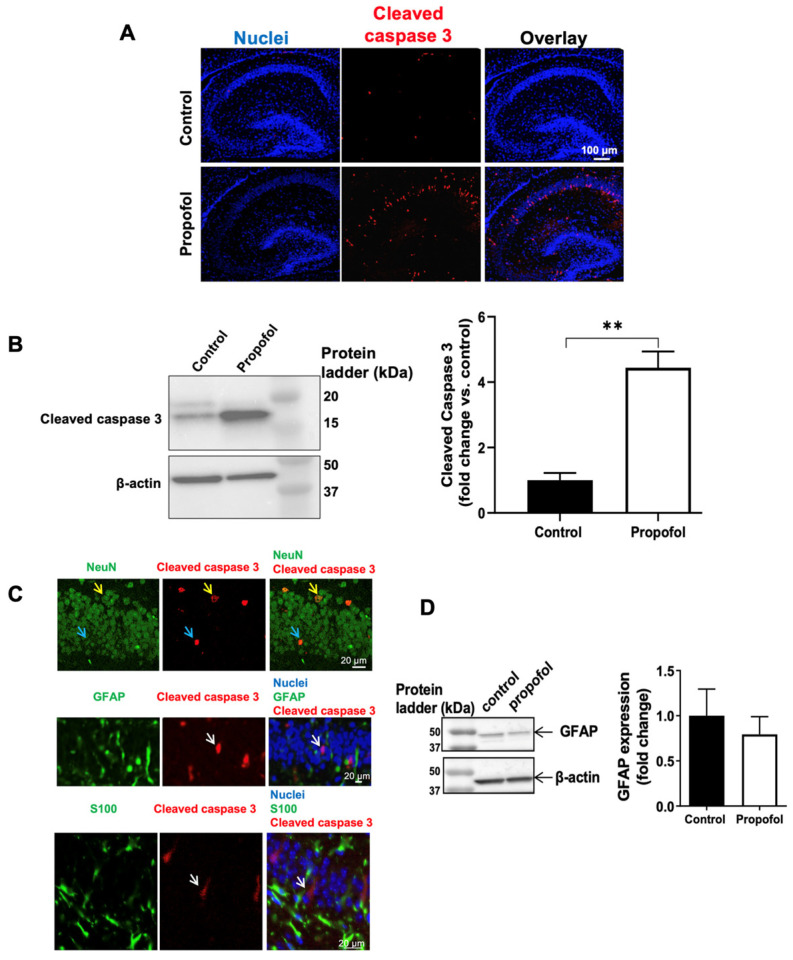
Neuroapoptosis in postnatal day 7 (P7) mouse hippocampi was induced by propofol. (**A**) Immunofluorescent staining and imaging showed that 6 h-propofol exposure at P7 induced acute cleaved caspase 3-positive apoptotic cells in mouse hippocampal tissue. Blue are cell nuclei and red are cleaved caspase 3-positive apoptotic cells. Scale bar = 100 µm. (**B**) The propofol-induced apoptosis was further confirmed and quantified by Western blot. n = 4, ** *p* < 0.01. (**C**) Propofol exposure led to apoptosis in neurons but not astrocytes in the hippocampus. The cleaved caspase 3-positive apoptotic signals (red) were located in neuronal nuclear antigen (NeuN; neuron marker)-positive neurons (green) but not in glial fibrillary acidic protein (GFAP) and S100 (astrocyte marker)-positive astrocytes. Blue are cell nuclei stained with Hoechst 33342. Two representative apoptotic neurons are indicated by yellow and blue arrows. Non-astrocyte apoptotic cells are indicated by white arrows. Scale bar = 20 µm. (**D**) Propofol exposure for 6 h did not alter the GFAP expression in hippocampal tissue. n = 4.

Briefly, P7 paraffin-embedded brain tissue blocks were cut into 4 µm-thick sagittal sections. The sections were then deparaffinized, hydrated, and subjected to antigen retrieval and washes with phosphate buffered saline (PBS), containing 0.5% Triton X-100 (Sigma-Aldrich, St. Louis, MO, USA) as previously described [38]. To identify which types of brain cells undergo apoptosis following propofol exposure, the sections were co-stained with the following primary antibodies: rabbit anti-cleaved caspase 3 (apoptosis marker; Cell Signaling Technology, Danvers, MA, USA, #9664; 1:200 dilution) along with either mouse anti-neuronal nuclear antigen (NeuN: neuron marker; MilliporeSigma, Burlington, MA, USA, MAB377; 1:100 dilution), goat anti-glial fibrillary acidic protein (GFAP: astrocyte marker; abcam, Waltham, MA, USA, ab53554; 1:200 dilution), and S100 (astrocyte marker; abcam, Waltham, MA, USA, ab868; 1:200 dilution) for one hour at 37 °C [41]. After 3 washes, slides were incubated with Alexa Fluor 488-conjugated donkey anti-mouse IgG or goat IgG together with Alexa Fluor 594-conjugated donkey anti-rabbit (Thermo Fisher Scientific, Waltham, MA, USA) for 45 min at 37 °C. After 3 more washes with PBS, the cellular nuclei were stained with Hoechst 33342 (Thermo Fisher Scientific, Waltham, MA, USA). The stained sections were imaged using Olympus Fluorescent Slide Scanner (Olympus Corporation of the Americas, Breinigsville, PA, USA). For positive control staining, we used mouse brain tissues. These antibodies resulted in the positive staining in mouse brain tissues, as shown in Figure 1. We also used induced pluripotent stem cells as negative control tissues for staining, showing no fluorescent signals using the same antibodies.

### 2.3. Western Blot

P7 brain tissues were collected and lysed in RIPA lysis buffer (Cell Signaling Technology, Danvers, MA, USA) supplemented with a phosphatase and protease inhibitor cocktail (Roche Diagnostics, Indianapolis, IN, USA) [42]. Lysates were centrifuged at 10,000× *g* for 10 min at 4 °C. Total protein concentration of the supernatants was determined using a DC Protein Assay Reagents Package kit (Bio-Rad, Hercules, CA, USA). The samples were boiled for 5 min. 25 μg of protein was loaded per lane for 4–20% sodium dodecyl sulfate polyacrylamide gel electrophoresis, and then transferred to nitrocellulose membrane. Membranes were blocked with blocking buffer (Thermo Fisher Scientific, Waltham, MA, USA) and incubated overnight at 4 °C with the following primary antibodies: rabbit anti-cleaved caspase 3 (Cell Signaling Technology, Danvers, MA, USA, #9664; 1:1000 dilution), mouse anti-GFAP (abcam, Waltham, MA, USA, ab10062; 1:1000 dilution), and rabbit anti-β-actin (Santa Cruz Biotechnology, Dallas, TX, USA, sc-47778; 1:1000 dilution). The primary antibodies were then washed out with Tris-buffered saline containing 0.1% Tween 20. Subsequently, the membranes were incubated with secondary antibodies conjugated to horseradish peroxidase (Cell Signaling Technology, Danvers, MA, USA) for one hour at room temperature and then with chemiluminescence detection reagent (Cell Signaling Technology, Danvers, MA, USA). The labeled proteins were imaged by using Chemidoc imaging system (Bio-Rad, Hercules, CA, USA). Optical densities of protein signals were quantified using Fiji ImageJ software (https://imagej.net/software/fiji/, accessed on 29 June 2022). The abundance of protein level was normalized with an internal control of β-actin.

### 2.4. Cognitive Function Assay

Morris water maze was used to determine spatial learning and memory of P60 mice receiving propofol exposure (6 h) at P7, as we described previously [43]. Briefly, a circular polypropylene pool (100 cm in diameter and 20 cm in height) was filled with water supplemented with a non-toxic white paint, rendering it opaque. On the pool rim, four points were designated (north, east, south, and west), dividing the pool into four quadrants (NE, NW, SW, and SE). The water was changed and its temperature was checked daily to be 20–22 °C. A platform (8 × 8 cm) was positioned at the center of the SE quadrant, with the standing area submerged ~2 cm below the surface of the water. Each mouse was tracked via EthoVision XT (Noldus Information Technology, Leesburg, VA, USA) starting from a random start point until it reached the platform or after one minute. If unable to find the platform in one minute, the mouse was guided by the investigator to reach the platform. Trials (learning test) were repeated 4 times (an interval of 5 min) per day for 5 days. On the 6th day, the platform was removed from the pool for testing the memory of mice. The mouse positioned in the water from a new start point was allowed to swim for one minute while tracked. Latency to escape was defined as the time that mice spent to find the platform during 5 days of trials or the platform zone on the 6th day.

### 2.5. Electrophysiological Assays

Control and propofol (6 h)-exposed P60 mice were decapitated. Hippocampi, the brain region related to various cognitive and behavioral functions [44,45], were embedded in 3% low-melting-point agarose (Sigma-Aldrich, St. Louis, MO, USA) as described [46]. Transverse hippocampal slices were cut at 400 μm thickness using a vibrating slicer (Leica VT1200s, Leica Biosystems, Deer Park, IL, USA) in the sucrose-based solution (4–6 °C) containing the following (in mM): 220 sucrose, 23 NaHCO_3_, 2.5 KCl, 1.25 NaH_2_PO_4_, 0.5 CaCl_2_, 7 MgSO_4_, 1.1 sodium ascorbate, 3.1 sodium pyruvate, and 10 glucose [47]. The slices were transferred to and stored in artificial cerebrospinal fluid (ACSF) containing (in mM): 119 NaCl, 3 KCl, 2 CaCl_2_, 1 MgCl_2_, 1.25 NaH_2_PO_4_, 23 NaHCO_3_ and 10 glucose at room temperature for at least 30 min prior to use. All solutions were saturated with 95% O_2_ and 5% CO_2_.

Whole-cell voltage-clamp recordings and extracellular recordings of field excitatory postsynaptic potential (fEPSP) assays were conducted using patch clamp amplifiers (Multiclamp 700B, AutoMate Scientific, Berkeley, CA, USA) under infrared-differential contrast interference microscopy. Data acquisition and analysis were performed using digitizers (DigiData 1440A and 1550B, Molecular Devices, San Jose, CA, USA) and software pClamp 10 (Molecular Devices, San Jose, CA, USA), respectively. Signals were filtered at 2 kHz and sampled at 10 kHz. For miniature excitatory postsynaptic potential EPSC (mEPSC) recording, Na^+^ channel blocker tetrodotoxin (TTX, 0.5 μM) was added into the ACSF to block action potentials, and GABA_A_ receptor blocker picrotoxin (50 μM) was freshly prepared and dissolved into the ACSF through sonication for ~10 min. Hippocampal CA1 pyramidal neurons were voltage clamped at −70 mV with an internal solution, consisting of the following (in mM): 140 K-gluconate, 5 KCl, 2 MgCl_2_, 10 HEPES, 0.2 EGTA, 4 Mg-ATP, 0.3 Na_2_GTP, and 10 Na_2_-phosphocreatine at pH 7.2 (with KOH). For miniature inhibitory postsynaptic potential (mIPSC) recording, Na^+^ channel blocker tetrodotoxin (TTX, 0.5 μM) was added into the ACSF to block action potentials. Glutamate receptor antagonists 6-cyano-7-nitroquinoxaline-2,3-dione (CNQX, 10 µM) and D-2-amino-5-phosphonovaleric acid (D-AP-5, 20 µM) were present in the ACSF throughout the experiments. Hippocampal CA1 pyramidal neurons were voltage clamped at −70 mV with an internal solution, consisting of the following (in mM): 80 K-gluconate, 60 KCl, 10 HEPES, 0.2 EGTA, 2 MgCl_2_, 2 Mg-ATP, 0.3 Na_2_GTP, and 10 Na_2_-phosphocreatine at pH 7.2 (with KOH). Series resistance (15–30 MΩ) was monitored throughout the recordings and data were discarded if the resistance changed by more than 20%. The fEPSPs recordings were made using glass pipettes filled with 1 M NaCl (1–2 MΩ), placed in the stratum radiatum of the CA1 region of the hippocampal slices. The fEPSPs were evoked by stimulating the Schaffer collateral/commissural pathway at 0.033 Hz with a bipolar tungsten electrode (WPI). Input–output curves were generated by plotting the fEPSP slope against the presynaptic fiber volley amplitude following incremental stimulus intensities [48]. The paired pulse ratio (PPR) was recorded at an intensity that induced ~40% of the maximal evoked response with 20, 50, 100, 200, and 400 milliseconds (ms) inter-pulse intervals [49]. The chemicals used in electrophysiological experiments (TTX, CNQX, D-AP5 and picrotoxin) were purchased from Tocris Bioscience (Ellisville, MO, USA). All other common chemicals were obtained from Sigma-Aldrich (Sigma-Aldrich, St. Louis, MO, USA). The data of mEPSCs and mIPSCs were analyzed using mini analysis (Synaptosoft, NJ, USA). The analysis of mEPSCs and mIPSCs were performed with cumulative probability plots [50,51]. The PPR was calculated by dividing the mean amplitude of the second EPSP by that of the first EPSP.

### 2.6. Microarray Assay of Messenger RNA (mRNA) Profiling

Our previous studies have shown that both 3 h- and 6 h-propofol induced acute neurotoxicity and long-term cognition dysfunction [35,38,40]. Thus, to dissect the potential molecular mechanisms of propofol-induced long-term cognitive dysfunction, we conducted microarray assay of mRNA profiling from 3 h propofol or intralipid control-exposed P60 mouse hippocampal tissues. The hippocampi from P60 mice were lysed in Qiazol reagent (Qiagen, Germantown, MD, USA) and the total RNA was extracted by using a phenol-chloroform method as we described [42]. Any possible contamination of genomic DNA was eliminated by using DNA-free^TM^ DNA remove kit (Thermo Fisher Scientific, Waltham, MA, USA). The RNA was then used for microarray assays. The box plot and scatter plot were used to assess the signal reproducibility. Mouse mRNA Expression Microarray V3.0 assay was performed by Arraystar Inc. (Rockville, MD, USA) for analysis of the expression of 24,881 mRNAs in the hippocampi. The RNA was synthesized to cDNA, hybridized to the microarray probes for fluorescence intensity scanning. *p* value was calculated using unpaired *t*-test, and false-discovery rate (FDR) was calculated from Benjamini Hochberg FDR. Fold Change was calculated using the absolute ratio of the normalized intensities between propofol and control conditions. Similar distribution of overall mRNA transcriptome in the two groups was illustrated in scatter plot. The significantly differentially expressed mRNAs (Supplementary Table S1) were defined by the expression level above ± 1.5-fold change and *p* < 0.05 (propofol vs. control) and shown in volcano plot and heatmap. The volcano plot is constructed by plotting the negative log of the *p* value on the y axis (base 10). The x axis is the log of the fold change between propofol and control groups. The results from microarray assay were further validated by using reverse transcription polymerase chain reaction (RT-PCR) assay as described.

### 2.7. RT-PCR

Two dysregulated mRNAs (Filip1 and Nsmf) were randomly selected, and further validated by using RT-qPCR to verify the authenticity of microarray assay data, as previously described [42]. Briefly, cDNA was synthesized by using RevertAid™ First Strand cDNA Synthesis Kit (Thermo Fisher Scientific, Waltham, MA, USA) from a total RNA of 2.5 μg (with mixed primer of oligo d(T) and random hexamer). In the qPCR assays, the cDNA, PowerUp™ SYBR™ Green Master Mix (Applied Biosystems, Foster City, CA, USA), primers (listed in Supplementary Table S4), and pure water (Qiagen, Germantown, MD, USA) were mixed for reaction. PCR triplicates were used, and qPCR reactions were performed by using the QuantStudio™ 6 Real-Time PCR machine (Applied Biosystems, Foster City, CA, USA). The specificity of the PCR reaction was checked with the melting curves of PCR product at the end of reaction. The mean cycle threshold (Ct) values from the PCR triplicates were used, and the raw data for mRNA expression were further normalized against endogenous control β-actin and finally analyzed by using 2^−ΔΔCt^ calculation.

### 2.8. Bioinformatic Analysis of Propofol-Induced Dysregulated Synaptic Genes and the Related Pathways/Functions

To determine the molecular mechanisms of propofol-induced long-term abnormal synapse activity and cognitive impairment, we used the ingenuity pathway analysis (IPA) tool (Qiagen, Germantown, MD, USA) to analyze the signaling/pathway of these dysregulated genes involved. IPA is a bioinformatics tool used for predicting disease mechanisms and canonical physiological signaling pathways of the differentially expressed genes between different conditions. To further dissect the contribution of synaptic gene signaling in AIDN, first, we defined the propofol-induced dysregulated synapse-related genes through the synaptic ontology (SynGO) database (https://www.syngoportal.org, accessed on 3 March 2022) [32]. The annotations in the database were based solely on published experimental evidence. The brain expressed background gene set was used to identify the enriched synaptic components from the propofol-induced dysregulated genes. The SynGo database includes the information of synaptic localization and function of ~1112 synaptic genes [52]. We then performed enrichment pathway analysis of differentially expressed synaptic genes using Metascape (http://metascape.org, accessed on 6 March 2022), as previously described [53]. Through the IPA tool, we further analyzed the signaling/pathway/networks of these dysregulated genes in development, activity, and function of neurons and synapses, and cognition, as well as their association with cognitive dysfunction and neurological diseases. The analysis of signaling pathways and networks of the dysregulated genes was conducted according to the known individual gene’s participation in established pathways, based on literature included in IPA database. We then obtained a collection of prediction of possible implications in central nervous system (CNS) development and function, behavior, and neurological diseases (with Fisher’s exact test *p* < 0.05 calculated in IPA database).

### 2.9. Statistic Analysis

All values described were expressed as the mean ± standard error (SE) of the mean. Sample size was decided based on the pilot data from our lab and previous similar studies [54,55]. For gene and apoptosis analysis, n = 4 was chosen, and n = 6–14 was chosen for behavior test and electrophysiology analysis. The statistically significant differences of the data between control and propofol treatment groups were analyzed by unpaired Student’s *t*-test using GraphPad Prism (version 9.0, San Diego, CA, USA). A level of *p* < 0.05 was considered to be statistically significant.

## 3. Results

### 3.1. Propofol Exposure Induces Acute Neuroapoptosis in Neonatal Mouse Hippocampi

Immunofluorescent staining and imaging showed that 6 h-propofol exposure at P7 induced acute cleaved caspase 3-positive apoptotic cells in mouse hippocampi (Figure 1A). The percentage of apoptotic cells was 0.13% and 1.96% in control and ethanol-treated hippocampi, respectively (*p* < 0.01). Western blot confirmed the apoptosis (Figure 1B). Additionally, the apoptotic and NeuN-positive neuronal signals were co-localized in the same cells (Figure 1C, yellow arrow). It has been shown that activation of astrocytes-promoted neuron death [56,57]. Increased GFAP was the marker of the astrocyte activation. To dissect the additional contribution of astrocytes to the propofol-induced acute neurotoxicity, we analyzed the effect of propofol exposure on the astrocyte activation. However, we did not find apoptotic signaling located in the GFAP-positive astrocytes (Figure 1C) and the GFAP expression determined by western blot was not altered by propofol (Figure 1D). These data suggest that (1) propofol induces apoptosis in mouse hippocampal tissues, and (2) propofol exposure results in acute apoptosis in neurons but does not cause the death and activation of astrocytes (Figure 1B).

### 3.2. Developmental Propofol Exposure Leads to the Impaired Learning and Memory in P60 Mice

**Figure 2:**
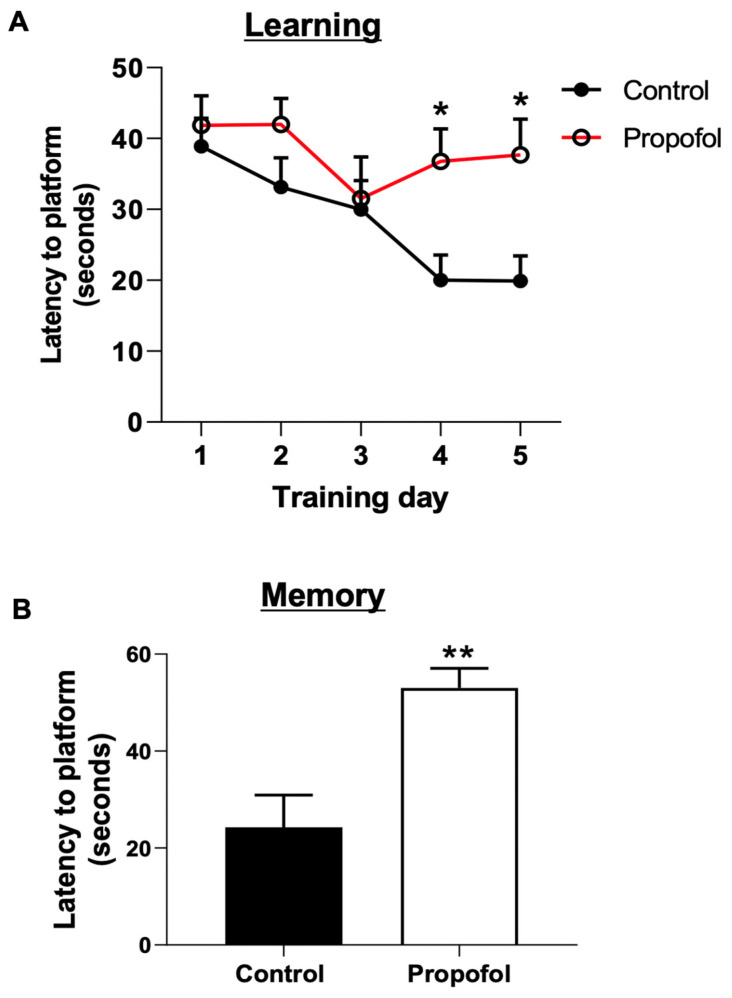
Morris water maze test revealed that P7 propofol exposure resulted in impairments in learning and memory in P60 mice. (**A**) The latency of propofol-exposed mice was longer than control mice at day 4 and 5 of learning tests. (**B**) The developmentally propofol-exposed P60 mice took longer time to find the platform at day 6 of memory test compared with control mice. n = 12, * *p* < 0.05, ** *p* < 0.01.

Mice received propofol or vehicle exposure at P7 (see Materials and Methods). The Morris water maze test was performed at P60 to assess spatial learning (first 5 days) and memory (6th day) in mice. The results showed that P7 propofol-exposed P60 mice displayed a longer time to find the platform in the water at day 4 and 5 of learning tests, compared with the intralipid treatment control mice, suggesting that 6 h-propofol exposure at P7 impaired the learning ability of P60 mice (Figure 2A). The propofol-exposed P60 mice also took longer time to reach the platform zone on the 6th day of the memory test) than control mice (Figure 2B), indicating the impaired memory of mice from the propofol group.

### 3.3. Propofol Exposure to P7 Mice Leads to the Disruption of E/I Balance in the Hippocampal Slices from P60 Mice

**Figure 3:**
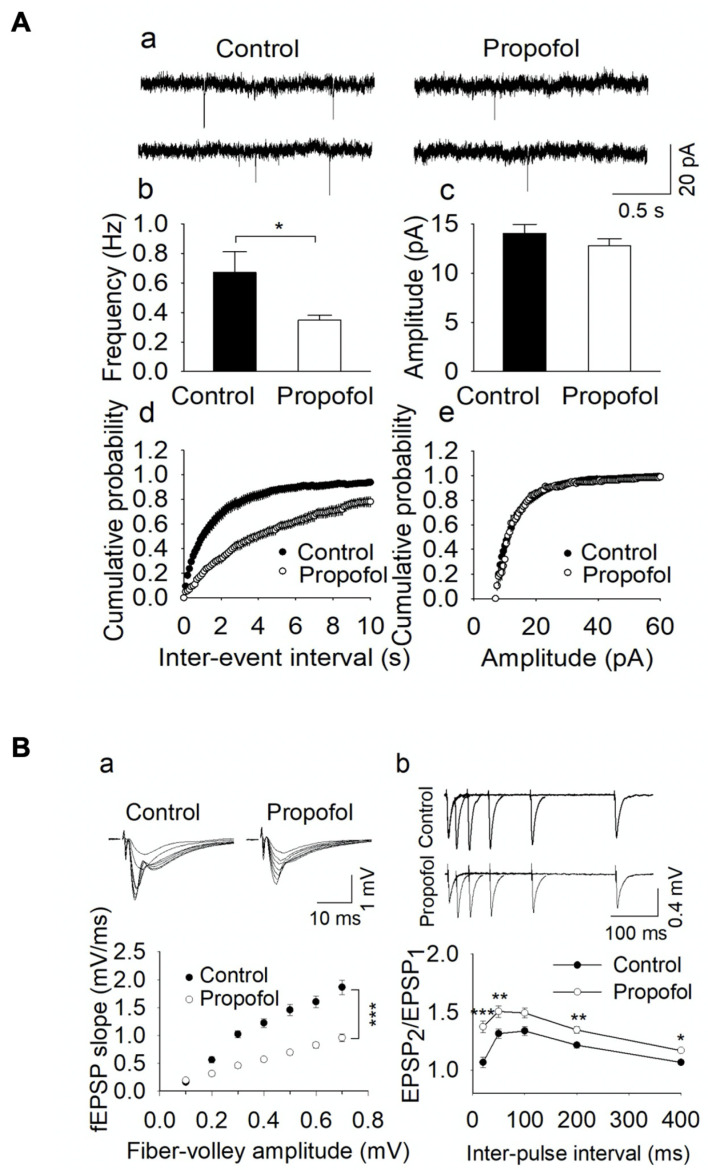
Developmental propofol exposure caused the decreased excitation/glutamate release in P60 mouse hippocampal neurons. (**A**) P7 propofol-exposed P60 mouse hippocampal CA1 pyramidal neurons displayed the reduction of excitation. (**a**) Examples of miniature excitatory postsynaptic current (mEPSC) traces from control and propofol groups. (**b**) The mean frequency of mEPSCs was decreased in propofol group (n = 13–14, *t*_25_ =2.356, * *p* < 0.05). (**c**) Propofol exposure did not alter the mean amplitude of mEPSCs (*t*_25_ = 1.126, *p* = 0.271). (**d**,**e**) Propofol resulted in a right shift of the cumulative distribution of inter-event intervals (n = 13–14; * *p* < 0.001). However, there was no difference of the cumulative amplitude distribution between control and propofol groups. Data from all events were averaged and pooled. (**B**) The field excitatory postsynaptic potential (fEPSP) slopes ((**a**), n = 6–7, *p* < 0.001) were decreased (**a**) and paired pulse ratio (PPR) was increased (**b**) by propofol ((**b**), n = 7; * *p* < 0.05, ** *p* < 0.01, *** *p* < 0.001).

To investigate the synaptic mechanisms of propofol-induced impairment of spatial learning and memory, we analyzed the E/I balance. Mice that received propofol or vehicle exposure at P7 and hippocampal slices were prepared from P60 mice. Whole-cell recordings were made in visually-identified CA1 pyramidal neurons in hippocampal slices. The mEPSCs were generated by action potential–independent quantal glutamate release from presynaptic axonal terminals. Changes in mEPSC frequency and amplitude indicate a presynaptic mechanism and postsynaptic responsiveness, respectively [58]. We found that developmental propofol exposure led to a significant decrease in the frequency of mEPSCs (n = 14 for control; n = 13 for propofol; *t*_25_ = 2.356, *p* = 0.027) (Figure 3(Aa,Ab)) but did not significantly affect the mean amplitude of mEPSCs (*t*_25_ = 1.126, *p* = 0.271; Figure 3(Ac)). Cumulative frequency plot analysis showed that developmental propofol exposure did not change the cumulative amplitude distributions (K-S test, *p* > 0.05; Figure 3(Ae)) but shifted the cumulative distribution of intervals between successive events to the right (i.e., longer intervals), consistent with the decrease in the frequency of mEPSCs (Figure 3(Ad)). These data suggest that developmental propofol exposure leads to a decrease in presynaptic glutamate release.

We next examined the input–output (I/O) relationships for fEPSPs by stimulating the Schaffer collateral/commissural pathway with incremental intensities. The fEPSP slopes were significantly decreased at all stimulation intensities with fiber volley amplitude greater than 0.2 mV in propofol group, compared with control group (*t*_11_ = 4.711, *p* < 0.001, Figure 3(Ba)). These results provide additional evidence that P7 propofol exposure resulted in the long-term decrease of basal excitatory transmission. To further distinguish between the presynaptic vs. postsynaptic effects, we recorded the paired-pulse fEPSPs at increasing inter-pulse intervals of 20–400 ms. A change in the paired-pulse ratio (PPR) suggests an alteration in presynaptic release probability [58]. Propofol exposure at P7 significantly led to an increase in the PPR at the inter-pulse intervals of 20 ms (*t*_12_ = 4.874, *p* < 0.001), 50 ms (*t*_12_ = 3.312, *p* = 0.006), 200 ms (*t*_12_ = 3.123, *p* = 0.009), and 400 ms (*t*_12_ = 3.005, *p* = 0.011), whereas the PPR at the inter-pulse interval of 100 ms (*t*_12_ = 2.147, *p* = 0.053) was not significantly changed (Figure 3(Bb)). The increase in the PPR was consistent with the decrease in the frequency of mEPSCs. Together, these results suggest that propofol exposure decreases presynaptic glutamate release.

**Figure 4:**
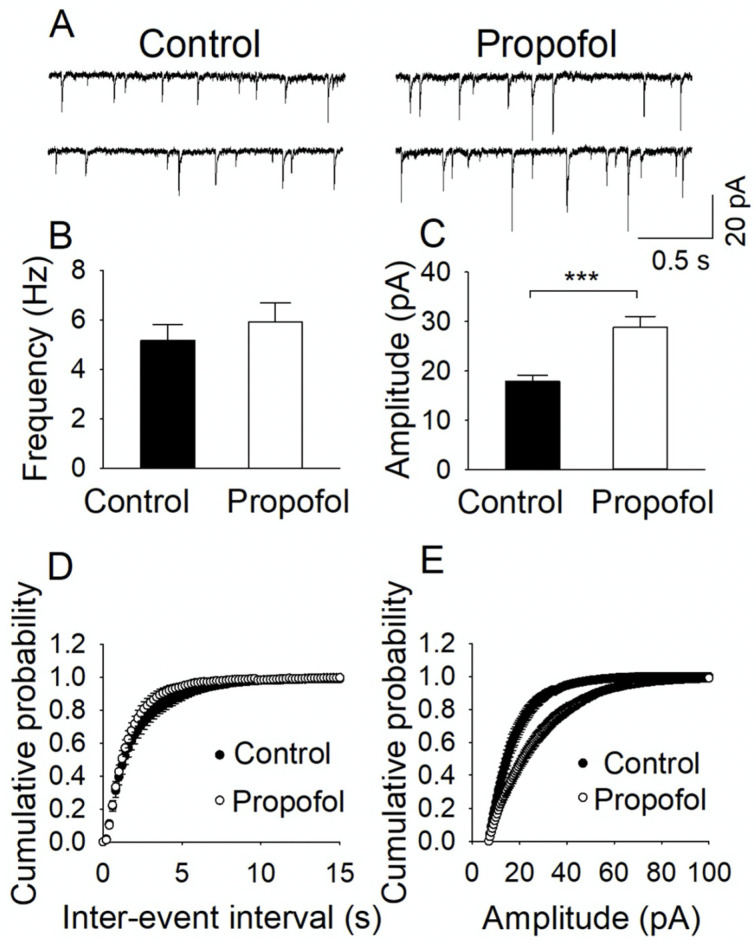
Propofol exposure led to an increase of inhibition in P60 mouse hippocampal neurons. (**A**) Example of miniature inhibitory postsynaptic current (mIPSC) traces. (**B**) The mean frequency of mIPSCs was not significantly changed in propofol group (n = 9–10; t_17_ = 0.766, *p* = 0.454). (**C**) Propofol exposure increased the mean amplitude of mIPSCs (t_17_ =4.380, *** *p* < 0.001). (**D**,**E**) Propofol did not influence the distribution of mIPSC inter-event interval values but resulted in a right shift of the cumulative amplitude distribution (n = 9–10; *p* < 0.01). Data from all events were averaged and pooled.

We further determined whether the propofol exposure at P7 altered inhibitory synaptic transmission in CA1 pyramidal neurons in the hippocampal slices prepared from P60 mice. The data showed that propofol exposure at P7 did not alter the mean frequency of mIPSCs (*t*_17_ = 0.766, *p* = 0.454) (Figure 4A,B) and inter-event intervals (Figure 4D). However, propofol-exposed mice displayed an increase of the mean amplitude of mIPSCs (n = 9, control; n = 10, propofol; *t*_17_ = 4.380, *p* < 0.001) (Figure 4C). The cumulative amplitude distribution was also shifted rightward in the propofol group (Figure 4E). These results indicate that developmental propofol exposure increases inhibitory synaptic transmission and this effect is likely mediated by an increase in postsynaptic responsiveness. The decrease in excitation and increase in inhibition suggest that developmental propofol exposure results in disruption E/I balance and long-term synapse dysfunction.

### 3.4. P7 Propofol Exposure Induces the Alteration of mRNA Profiles in P60 Mouse Hippocampi

**Figure 5:**
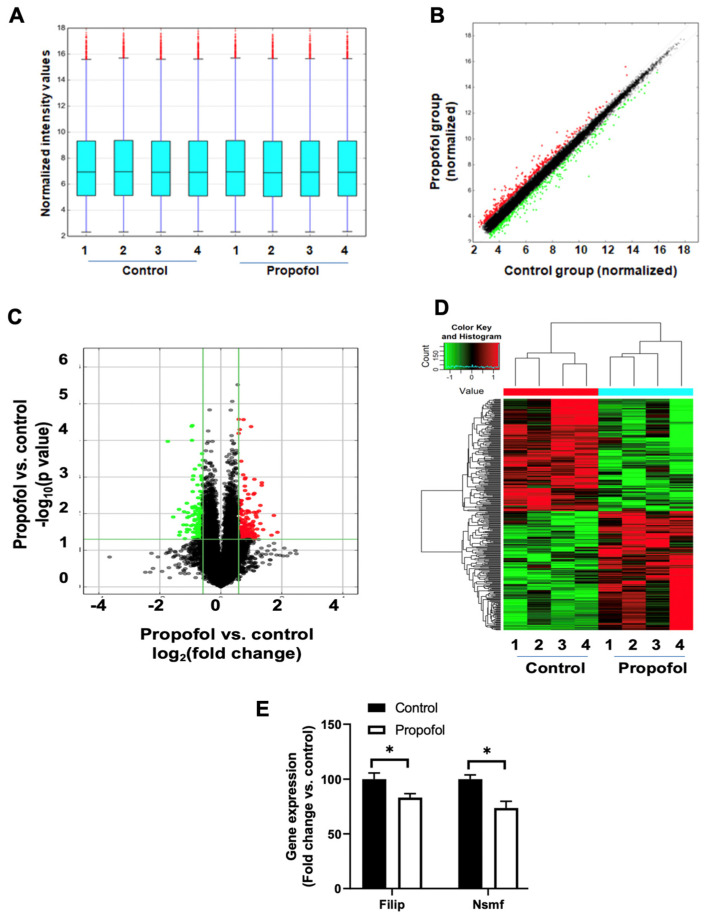
Developmental propofol exposure induced the differential expression of mRNA profiles in the P60 mouse hippocampi. (**A**) The box plots displays the similar distribution of normalized mRNA signal intensity values from 4 control and 4 propofol-treated mouse hippocampal tissues. For each box, the central line represents the median of mRNA intensity values, whereas the tails represent the upper and lower quartiles. (**B**) The scatter plot shows the general consistence of normalized mRNA intensity values from control and propofol groups. (**C**) The volcano plot illustrates the abnormally expressed 317 mRNAs between control and propofol groups. (**B**,**C**) Red dots: up-regulated mRNAs; green dots: down-regulated RNAs (n = 4, *p* < 0.05, fold change ≥ 1.5, propofol vs. control). The significant gene names were listed in the Supplementary Table S1. (**D**) Heatmap and hierarchical clustering displays the expression profiles of propofol-induced differentially expressed mRNAs in mouse hippocampi (*p* < 0.05). Each row represents the relative expression of each gene. (**E**) RT-PCR validation of the expression of two randomly selected dysregulated mRNAs from microarray assay. n = 4, * *p* < 0.05.

To further investigate the underlying molecular mechanisms of propofol-induced synapse dysfunction and impaired learning and memory capacity, we used an unbiased approach of the microarray assay to evaluate the expression profiles of 24,881 mRNA transcripts. The distribution of normalized microarray intensity values of each sample was displayed in a box plot showing the spread and centers of a data set (Figure 5A), indicating the similar distribution of the data from control and propofol groups, and the data are suitable for further analysis. The scatter plot showed that the normalized expression data were highly consistent between control and propofol groups (person correlation: 0.995) (Figure 5B). Among 24,881 mRNAs analyzed, propofol exposure resulted in 317 dysregulated mRNAs (163 upregulated and 154 downregulated) (fold change above ±1.5, *p* < 0.05) in P60 hippocampal tissues. The volcano plot and heatmap illustrated the differentially abundant mRNAs in the hippocampi between control and propofol groups (Figure 5C,D). The list of all dysregulated genes and their full gene name were included in Supplementary Table S1. The mRNA expression level of randomly selected altered two genes were further validated using reverse transcription-quantitative polymerase chain reaction (Figure 5E) and the data were consistent with what was obtained from the microarray assay.

We then used IPA to analyze the functions/pathways/diseases of the propofol-induced abnormally expressed 317 mRNAs. These dysregulated genes have been previously reported to be involved in various cellular biology functions (e.g., cellular homeostasis, cell movement, migration of neurons, and synaptic vesicle cycle), neuronal injury (e.g., damage of hippocampus), and neurological diseases (e.g., AD, encephalopathy, and brain lesion). For instance, among these 317 propofol-induced dysregulated genes, 8 genes were related to ephrin receptor signaling, 9 genes were related to migration of neurons, 26 genes were associated with neuronal cell death and damage of hippocampus, and 69 genes involved in various neurological diseases (e.g., encephalopathy, AD, and brain lesion) (Supplementary Figure S1). Notably, the ephrin receptor signaling has been shown to be important in the regulation of synapse formation, function, and plasticity [59].

### 3.5. Propofol Induces Dysregulation of Signaling Pathways Related to Synaptic Activity and Cognitive Dysfunction

**Figure 6:**
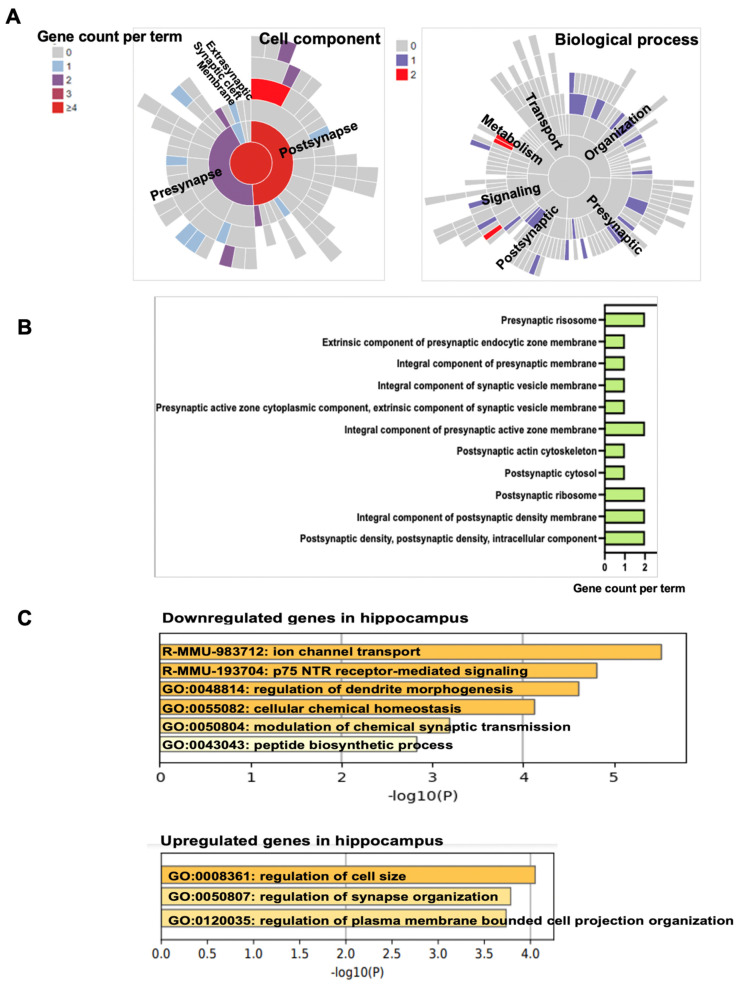

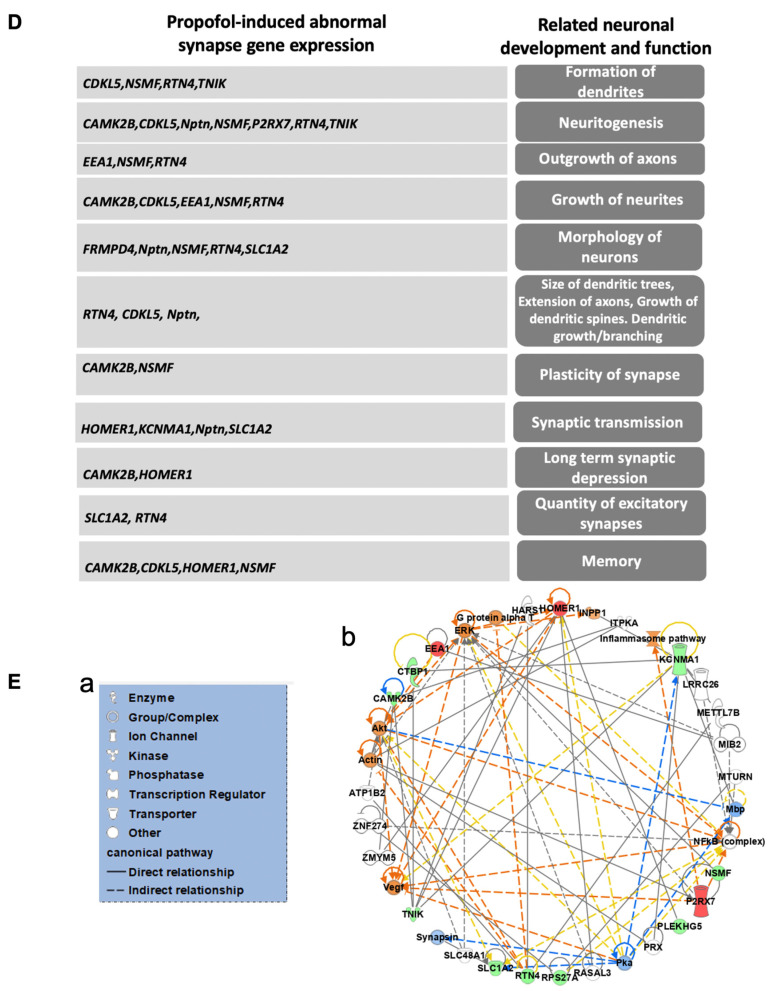
Propofol−induced dysregulated synaptic genes in P60 mouse hippocampi. (**A**) Bioinformatic analysis of 317 propofol−induced differentially expressed genes to define the dysregulated synaptic genes through the SynGO database. The analysis showed that 23−propofol−induced dysregulated genes were synaptic genes. The sunburst plot depicts the synapse location (cell component for presynapse, postsynapse, synaptic cleft, extra-synaptic space, or synaptic membranes) and functions (biological process related to metabolism, transport, synapse organization, synaptic signaling, presynapse, and postsynapse) of these 23 synaptic genes. Different color represents the gene counts per term of each location (**a**) or each function of synapse (**b**). The information of the synapse location and function of these 23 genes were described in Table 1 in detail. (**B**) The horizontal column chart depicts the gene count per synaptic cellular component shown in (**A**). (**C**) Gene ontology (GO) analysis of molecular functions of both downregulated and upregulated synaptic genes. (**D**) IPA bioinformatic analysis of function associated with propofol-induced dysregulated synaptic genes. Analysis revealed that the propofol−induced dysregulated synapse gene were associated with signaling, neuronal development and function, synaptic activity, and memory. The involvement of these synaptic genes in behavior was described in the Table 2. (**E**) The mechanistic regulatory networks of the propofol-induced dysregulated synaptic genes were predicted by network analysis using IPA. (**a**) Defining various nodes and lines depicted in Figure 6(Eb) circular molecular network. Each symbol represents one individual gene category, such as enzyme and ion channel. Solid and dotted lines show a direct and indirect connection between genes. (**b**) The predicted networks of the propofol−induced dysregulated synaptic genes. Gene names are shown on the molecular networks graph. Green symbols indicate downregulation and red indicate upregulated genes in propofol group vs. control. The abbreviations of the dysregulated synaptic genes were defined in Table 1.

Synaptic genes are genes directly involved in synapse function, as well as learning and memory. Therefore, we narrowed down our bioinformatic analysis from the 317 dysregulated genes to synaptic genes. We found that 23 abnormally expressed genes (14 downregulated/9 upregulated) were the annotated synaptic genes in SynGO database. Most of these dysregulated synaptic genes are located in presynapse and postsynapse (Figure 6A,B). These genes are related to (1) synapse cytoskelton structure/maturation/organization, (2) synaptic transmission and receptor activity, (3) presynapse and postsynapse to nucleus signaling pathway, and (4) synaptic vesicle trafficking (Table 1). Gene ontology (GO) (one of the main resources of biological information providing a specific definition of protein functions) analysis of downregulated or upregulated synaptic genes showed that molecular functions of these genes are involved in ion channel transport, p75 NTR receptor-mediated signaling, regulation of dendrite morphogenesis, cellular chemical homeostasis, modulation of chemical synaptic transmission, peptide biosynthetic process, and regulation of cell size/synapse organization/plasma membrane-bounded cell projection organization (Figure 6C).

We further used the IPA tool to analyze the functions, pathways, diseases, and regulatory networks associated with these synaptic genes. The analysis revealed that these dysregulated synaptic genes were related to neuronal activity, neuron development (e.g., formation of dendrites, neurogenesis, branching of neurites, size of dendritic trees, extension of axons, growth of dendritic spines), synapse formation, and synapse plasticity (Figure 6D). Additionally, many of these genes has been connected to cognition and behavior, such as conditioning, contextual conditioning, social withdrawal, working memory, nest building behavior, learning, object recognition memory, chaining behavior, anxiety, tone fear conditioning, and despair behavior (Table 2). Network analysis suggests that some of these synaptic genes may form regulative signaling networks (Figure 6E), thereby contributing to the impaired cognition and synapse deficits observed in propofol-treated mice. Further IPA bioinformatic analysis associates the propofol-induced decreased synaptic genes with various nervous system development and brain functions (Supplementary Table S2), as well as many neurological disorders (e.g., cognitive impairment, intellectual disability, stroke, and depression) (Supplementary Table S3).

## 4. Discussion

In this study, we studied propofol-induced acute and chronic developmental neurotoxicity in the aspects of pathology changes, synaptic activity, cognitive dysfunction, and alterations of global gene and synaptic gene expression profiles. We found that propofol-induced acute neuroapoptosis. Early life propofol exposure resulted in persistent impairment of learning and memory, as well as E/I imbalance, as evidenced by the decreased excitation and increased inhibition in the CA1 pyramidal neurons in the hippocampus. In lines with these long-lasting effects, propofol-exposed P60 hippocampi exhibited abnormal expression of synaptic genes. Further multiple bioinformatic analyses revealed the importance of these synaptic genes in synaptogenesis, synaptic activity and plasticity, and cognitive impairment associated with anesthetic neurodegeneration.

Both intravenous (e.g., ketamine) and volatile anesthetics (e.g., sevoflurane) could induce acute developmental neurotoxic effects, such as apoptosis in animal models [60]. Neurons were more vulnerable to propofol for apoptotic response than astrocytes (Figure 1). Similar neuron vulnerability to apoptosis was observed in the cultured rat hippocampal neurons and stem cell-derived human neurons [36,61,62]. We have shown that insufficient brain-derived neurotrophic factor (BDNF) secretion from astrocytes was involved in the increased propofol-induced rat hippocampal neuron apoptosis in cell culture models [36]. Astrocytes play various functions within the CNS, such as maintenance of molecular, cellular, and metabolic homeostasis, to regulate cognition and behavior. Astrocytes were also implicated in various neurological disorders. Astrocytes became activated in response to various brain injuries and diseases and the activated astrocytes lost the ability to promote neuronal survival [63]. To dissect the additional contribution of astrocytes to the propofol-induced acute neurotoxicity, we analyzed the effect of propofol exposure on the astrocyte activation. We found that GFAP expression in astrocytes was not affected by propofol, suggesting that astrocytes were not activated by propofol exposure and astrocyte activation did not contribute to the neuroapoptosis observed. However, how propofol-induced neuroapoptosis results in the long-term intellectual disability is largely unknown. This form of neurotoxic insult could dispute normal neurodevelopment with the potential to permanently shape cognitive ability and behavior.

It is important to identify cellular and molecular mechanisms underlying the anesthetic-induced chronic cognitive deficits as we (Figure 2) and other observed [4,8] showed that anesthetic-induced behavior changes and neuronal death. Anesthetic exposure led to suppressed long-term potentiation, long-term abnormal changes in synaptic structure and function, dendritic branches, total dendritic length, and the density of dendritic spines [64,65,66,67,68]. For instance, Sanchez et al. [66] showed that following anesthesia (isoflurane, nitrous oxide, and midazolam) exposure, the survived neurons exhibited long-lasting disturbance in inhibitory synaptic transmission in rat hippocampi. Zhou et al. [8] found that the activity of local inhibitory interneuron networks was altered by multiple exposures to propofol. Vasoactive intestinal peptide-expressing interneurons were hyperactive when the mice were performing a motor learning task. Thus, abnormal synapse structure and activity might be one of major mechanisms of anesthetic-induced impaired cognitive dysfunction. We found that developmental propofol exposure induced E/I imbalance in hippocampal CA1 pyramidal neurons, as shown by the increased inhibition and decreased excitation, supporting the adverse effect of developmental anesthetic exposure on the synaptic function (Figure 3 and Figure 4). The decreased excitation was resulted from the reduction of presynaptic glutamate release, while the increased inhibition was mediated by an increase in postsynaptic responsiveness, suggesting both presynaptic and postsynaptic mechanisms contributed to the E/I imbalance. So far, there is no defined connection between neuronal apoptosis and neuron release probability. Neuronal apoptosis might result in the malplasticity of surviving neurons and other types of brain cells. Neurons that survive anesthesia treatment might also be directly adversely influenced and have maladaptive plasticity, contributing to the long-term abnormal brain development, eventually leading to synaptic release change. The maintenance of E/I balance is important for appropriate synapse function, and dysregulation of interplay between excitation and inhibition have been linked to cognitive decline and abnormal behaviors [27,28,29,30,69,70,71]. Thus, the propofol-induced E/I imbalance might disrupt information processing and storing, thereby leading to the impairment of learning and memory. The deficits of synapse function and abnormal synaptic transmission might be the common mechanisms of different anesthetic-induced impaired cognition. So far, there is no defined connection between propofol-induced acute neuroapoptosis and abnormal long-term neuron release probability. Neuroapoptosis might result in the malplasticity of surviving neurons and other types of brain cells. Neurons that survive anesthesia treatment might also be directly influenced by propofol exposure, resulting in abnormal gene expression profiles and molecular signaling, maladaptive plasticity, synaptic release change, and cognitive deficits.

Abnormal expression or mutations of the synapse function-related genes have been connected to neurodevelopmental disorders and intellectual disability (e.g., autism, Fragile X syndrome, and AD) [31,72]. Thus, the long-term neurotoxic effects of propofol on synaptic activity and cognitive function could be resulted from the alterations of synapse function-related gene expression (Table 1), as well as molecular signaling and networks (Figure 6D,E and Figure S2). Consistent with our E/I balance data, propofol-induced changes in both presynaptic and postsynaptic genes, which are critically involved in synaptic function and CNS development and function. For instance, propofol exposure resulted in the downregulation of potassium calcium-activated channel subfamily M alpha 1 (*KCNMA1*). The *KCNMA1* encodes the BK channel, an integral component of presynaptic active zone membrane, and regulates presynaptic membrane potential and synaptic transmission. The gain-of-function mutation of KCNMA1 led to faster repolarization of action potential and increased neuronal excitability [73]. Another decreased gene is TRAF2 and NCK interacting kinase (*TNIK*). The TNIK is a post-synaptically-enriched protein and important for cognitive function through synaptic and nuclear signaling pathways. The knockdown of TNIK in primary cultured neurons altered the synchrony of network activity [74,75]. C-terminal binding protein 1 (CtBP1) is a ubiquitous regulator of membrane trafficking. The propofol-exposed mouse hippocampi displayed the reduced *CtBP1* expression. The deficiency of *CtBP1* led to defects in synaptic vesicles retrieval and synaptic depression during sustained neurotransmission. Thus, the alterations of the synapse gene expression profiles, such as the downregulation of KCNMAS, TNIK, and CtBP1, might explain the molecular bases for the propofol-induced changes of presynaptic glutamate release and postsynaptic responsiveness. It is possible that these dysregulated genes did not independently contribute to the synaptic dysfunction and cognitive dysfunction. The 23 propofol-induced dysregulated genes were located in the different synaptic compartments and each gene had their distinctive roles in comprising the synapse (Table 1 and Figure 6A–C), such as action potential, synapse organization, maturation, synapse transmitter reuptake, synaptic transmission, signaling, and synaptic vesicle endocytosis. Some of these genes were predicted to form signaling networks (Figure 6D,E and Figure S2). Thereby, it is possible that the alteration of multiple synaptic gene expression/network might result in the adverse remodeling of synapse architecture and signaling, leading to an abnormal synapse transmission, information processing and storage, and impaired cognitive function.

Although the focus of our data analysis was on the dysregulated synaptic genes, we cannot exclude the potential roles of other propofol-induced non-synapse-related genes (Figure 5 and Supplementary Table S1) in the neurotoxicity. For instance, IPA bioinformatic analysis showed that eight propofol-induced dysregulated genes (*CXCL12*, *EFNA1*, *ERAS*, *GNAS*, *GRINA*, *ITGAL*, *PDGFD* and *RGS3*) that are not included in the SynGO database were associated with ephrin receptor signaling (Supplementary Figure S1). Ephrin receptor signaling has been shown to be involved in the storage of memory by the regulation of events, such as transmitter release and reuptake, and synaptogenesis. IPA analysis revealed the molecular network between some of these dysregulated ephrin receptor signaling genes and synaptic genes (Supplementary Figure S2), suggesting the complex singling networks underlying the propofol-induced synaptic and cognitive dysfunction. However, the precise roles of these dysregulated ephrin receptor signaling genes in AIDN remain to be examined. Additionally, whether different anesthetics share similar molecular mechanisms in AIDN remain to be determined.

Collectively, our studies suggest that the E/I imbalance may underlie developmental propofol exposure-induced long-term impairment of learning and memory. The use of powerful unbiased transcriptomic and in-depth bioinformatic analysis further sheds light on the molecular mechanistic basis of the synaptic deficits and cognitive dysfunction. Importantly, the findings provide a valuable insight into the novel strategies for the therapeutic intervention of AIDN, including manipulations of gene expression/signaling and restoring E/I balance.

## Figures and Tables

**Table 1:** The cellular location and functions of propofol-induced dysregulated synaptic genes.

Gene Symbol	Gene Name	Location of Cellular Component	Related Biological Process of Synapse	Expression Change (Propofol vs. Control)
***ADRBK1***	G protein-coupled receptor kinase 2	postsynaptic density, presynapse		down
***ATP6V0C***	ATPase H+ transporting V0 subunit c	integral component of synaptic vesicle membrane		down
***ATP6V1G1***	ATPase H+ transporting V1 subunit G1	extrinsic component of synaptic vesicle membrane	synaptic vesicle proton loading	down
***CAMK2B***	calcium/calmodulin dependent protein kinase II beta	postsynaptic density	regulation of synapse maturation, structural constituent of postsynaptic actin cytoskeleton	down
***CTBP1***	C-terminal binding protein 1	presynaptic active zone cytoplasmic component, extrinsic component of presynaptic endocytic zone membrane	synaptic vesicle endocytosis, synaptic vesicle clustering, synaptic vesicle clustering, presynapse to nucleus signaling pathway	down
***FILIP1***	filamin A interacting protein 1	postsynapse, postsynaptic actin cytoskeleton	modification of postsynaptic structure	down
***KCNMA1***	potassium calcium-activated channel subfamily M alpha 1	integral component of presynaptic active zone membrane	ligand-gated ion channel activity involved in regulation of presynaptic membrane potential	down
***NSMF***	NMDA receptor synaptonuclear signaling and neuronal migration factor	postsynapse	postsynapse to nucleus signaling pathway	down
***Plekhg5***	pleckstrin homology domain containing, family G (with RhoGef domain) member 5		pleckstrin homology and RhoGEF domain containing G5	down
***RPL38***	ribosomal protein L38	postsynaptic density, synapse, postsynaptic ribosome, presynaptic ribosome	translation at presynapse	down
***RPS27A***	ribosomal protein S27a	synapse, postsynaptic ribosome	translation at presynapse, translation at postsynapse	down
***RTN4***	reticulon 4	postsynapse, integral component of postsynaptic density membrane	modulation of chemical synaptic transmission	down
***SLC1A2***	solute carrier family 1 member 2	integral component of presynaptic membrane	neurotransmitter reuptake	down
***TNIK***	TRAF2 and NCK interacting kinase	presynapse, postsynaptic density, intracellular component	regulation of neurotransmitter receptor localization to postsynaptic specialization membrane	down
***ANO6***	anoctamin 6	integral component of synaptic membrane	regulation of postsynaptic membrane potential	up
***CDKL5***	cyclin dependent kinase like 5	postsynaptic density, intracellular component	modulation of chemical synaptic transmission, regulation of postsynapse organization	up
***EEA1***	early endosome antigen 1	postsynapse	postsynaptic process involved in chemical synaptic transmission	up
***ELAVL2***	ELAV like RNA binding protein 2	synapse	regulation of synapse assembly	up
***FRMPD4***	FERM and PDZ domain containing 4	postsynaptic density	postsynaptic actin cytoskeleton organization	up
***HOMER1***	homer scaffold protein 1	postsynaptic density, postsynaptic cytosol	regulation of postsynaptic neurotransmitter receptor activity, structural constituent of postsynapse	up
***NPTN***	neuroplastin	synaptic membrane, integral component of presynaptic active zone membrane, integral component of postsynaptic density membrane	trans-synaptic signaling by trans-synaptic complex, modulating synaptic transmission, trans-synaptic signaling by trans-synaptic complex, modulating synaptic transmission, postsynapse	up
***P2rx7***	purinergic receptor P2X, ligand-gated ion channel, 7		purinergic receptor P2X 7	up
***TDRD6***	tudor domain containing 6	synapse		up

**Table 2:** Propofol-induced dysregulated synaptic genes associated with cognition and behavior.

Molecules	Diseases or Functions Annotation
CAMK2B,CDKL5,HOMER1,KCNMA1,NSMF	Conditioning
CAMK2B,CDKL5,NSMF	Contextual conditioning
CDKL5,RTN4	Social withdrawal
CDKL5,HOMER1	Working memory
CAMK2B,CDKL5	Nest building behavior
CAMK2B,CDKL5,HOMER1,NSMF,RTN4	Learning
CAMK2B,CDKL5,HOMER1,NSMF	Memory
CAMK2B,NSMF	Object recognition memory
P2RX7	Locomotion of vesicles
HOMER1	Chaining behavior
HOMER1,KCNMA1,P2RX7,RTN4	Locomotion
HOMER1	Lever press response
HOMER1	Navigation
RTN4	Perseverance behavior
CAMK2B,CDKL5,HOMER1	Anxiety
HOMER1	Tone fear conditioning
HOMER1	Cocaine seeking behavior
KCNMA1	Blinking
CAMK2B	Hippocampal learning
KCNMA1	Eyeblink conditioning
P2RX7	Coping response
HOMER1	Habituation
HOMER1	Despair behavior
KCNMA1	Swimming behavior
KCNMA1	Circling behavior

## Data Availability

All data generated or analyzed in this study are included in the manuscript and Supplementary Materials.

## Supplementary Materials

The following supporting information can be downloaded at: https://www.mdpi.com/article/10.3390/cells11162497/s1, Figure S1: Bioinformatic analysis shows cellular biology, neurological diseases, and signaling pathways related to the propofol-induced dysregulated genes; Figure S2: Bioinformatic analysis predicts the molecular networks between propofol-induced dysregulated ephrin receptor signaling genes and synaptic genes; Table S1: Propofol-induced dysregulated mRNAs; Table S2: Propofol-induced dysregulated synapse genes involved in nervous system development and function; Table S3: Propofol-induced downregulated synapse genes related to neurological disorders; Table S4: Sequence information for primers.
