# Supplementary material for: Integrated Excitatory/Inhibitory Imbalance and Transcriptomic Analysis Reveals the Association between Dysregulated Synaptic Genes and Anesthetic-Induced Cognitive Dysfunction
Source: Cells. 2022 Aug 11;11(16):2497. doi: 10.3390/cells11162497 (PMC9406780; doi:10.3390/cells11162497)
Supplement: Supplementary file 1 [file cells-11-02497-s001.zip › Supplementary Figures S1 and S2 .pdf]

Supplementary Figure S1

Propofol-induced abnormally expressed genes	Related diseases, signaling, and cellular biology
CXCL12, EFNA1, ERAS, GNAS, GRINA, ITGAL, PDGFD, RGS3	Ephrin receptor signaling
AGA,ALB,ATP6V1G1,CAMLG,CD247,CHMP4C,CRP,CXCL12,CYBRD1,ENG,GAB1,GLI3,Igtp,INHBA,ITGAL,KCNMA1,KLF10,LEPR,Nptn,P2RX7,PCSK1,PECAM1,Pln,PTPRB,RAPGEF3,RTN4,TCF12,THBS4,TNFAIP3,TYK2,ZDHHC7	Cellular homeostasis
ALB,ANO6,CARD14,CCL22,CDK1,CDKL5,CLDN5,CRH,CRP,CRYAB,CXCL12,CXCR5,CXXC5,DAB2,DDT,EBF2,EFNA1,ENG,FLNB,FOXG1,GAB1,GLI3,GNAS,HTR6,ITGAL,KCNMA1,KLK3,LAMC2,LEPR,LGMN,LGR6,MTA1,MYO1C,MYO1F,NDN,NFIB,OVOL2,P2RX7,PDLIM1,PECAM1,PLEKHG5,Prl2c2,PROM1,PTPRB,RAPGEF3,RTN4,SKAP1,SLC1A2,SNAI1,SSH2,TBX1,THBS1,THBS4,TNIK,TSPAN32	Cell movement
CAMK2B,CAMLG,CDK1,CXCL12,DAB2,DYNC1I1,EME1,FOXG1,GAB1,GLI3,HK2,KLK3,MTA1,NDNF,NPNT,PCDHGC5,RBPJ,SLC1A2,SNAI1,TNFAIP3,TOP2A	Cell survival
ATP6V0C/ATP6V1G1/SLC1A2D	Migration of neurons
AGA,ATF3,CAMK2B,CDC25B,CDK1,CXCL12,DYNC1I1,EPHX2,FOXG1,GLI3,HK2,LEPR,INHBA,INPP5A,KCNMA1,LGMN,MAP3K5,NDNF,NSMF,P2RX7,PCDHGC,RAPGEF3,SLC1A2,THBS1,TNFAIP3,TYK2	Neuronal cell death/Damage of hippocampus
ACBD5,ACSS3,AGA,AKAP13,ATF3,BRD8,CDC73,CDCA2,CDK12,CDKL5,CLSPN,DAB2,EBF2,ECSIT,EEA1,EPHX2,FLNB,FRMPD4,GLI3,GOLIM4,HOMER1,HSDL1,INHBA,ITGAL,KHDC3L,KIF1B,KLF10,LAMC2,LEPR,LIPG,MAP4,MBNL1,MDN1,MYO15A,MYOF,NDNF,NFIB,NSMF,NUSAP1,P2RX7,PAPPA2,PCSK1,PDGFD,PRKRIP1,RAB37,RAPGEF3,RTN4,SCIN,SERINC2,SLC1A2,SLC24A5,SPPL3,SPTBN4,STOX1,STRIP2,STYK1,TBX1,TCF12,TDRD6,THBS1,THBS4,TNFAIP3,TNIK,TOP2A,TRIP11,TRPM3,TTC37,WNT9B,ZDHHC7	Neurological diseases (e.g., cerebral disorders, encephalopathy, Alzheimer disease, Brain lesion）

Supplementary Figure S2

A

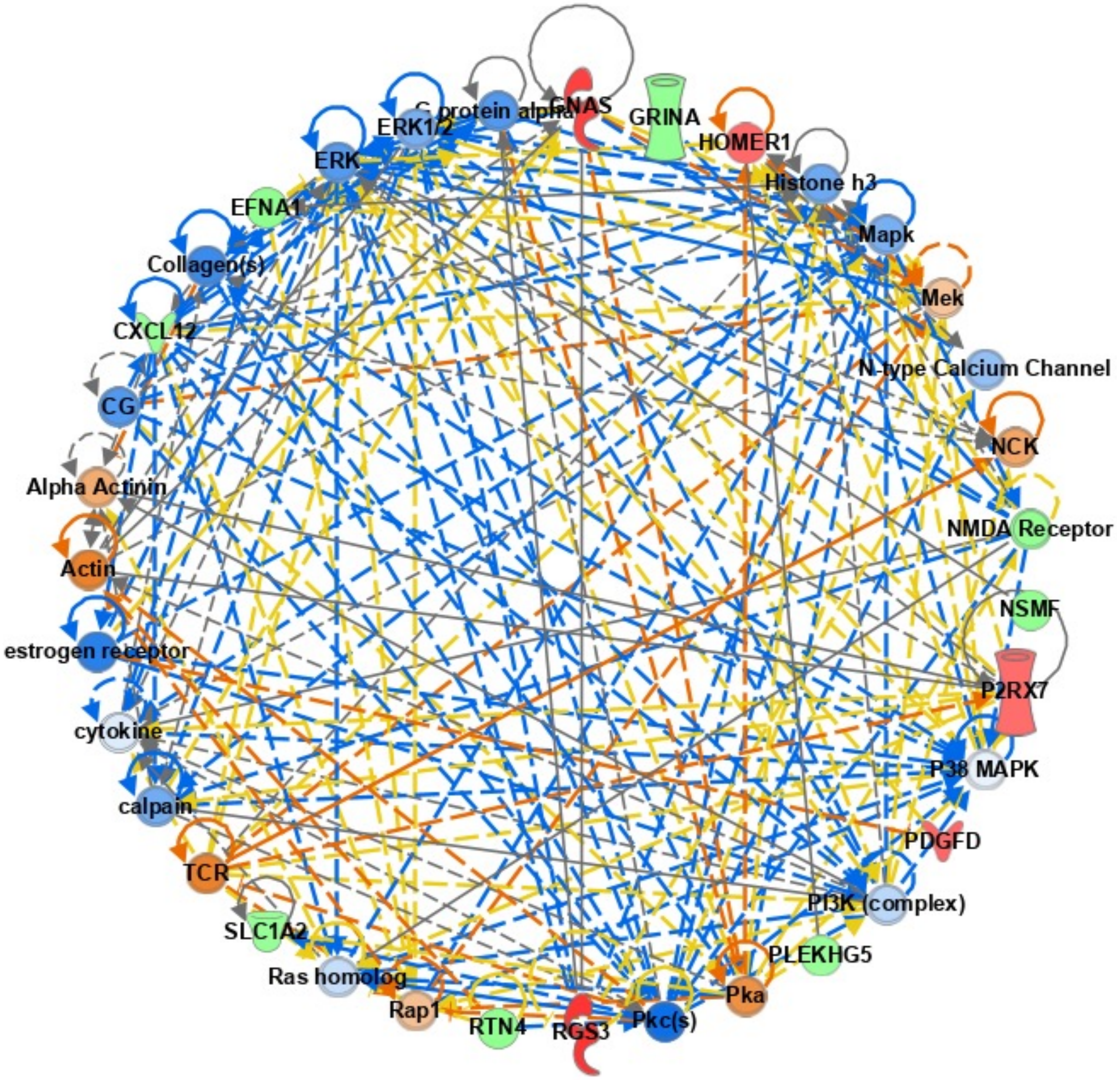

B

Actin	Alpha Actinin	calpain	CG
Collagen(s)	**CXCL12**↓	cytokine	**EFNA1**↓
ERK	ERK1/2	estrogen receptor	G protein alphai
**GNAS**↑	**GRINA**↓	Histone h3	**HOMER1**↑
Mapk	Mek	N-type Calcium Channel	NCK
NMDA Receptor	**NSMF**↓	**P2RX7**↑	P38 MAPK
**PDGFD**↑	PI3K (complex)	Pka	Pkc(s)
**PLEKHG5**↓	Rap1	Ras homolog	**RGS3**↑
**RTN4**↓	**SLC1A2**↓	TCR	

Supplementary Figure 1 Bioinformatic analysis shows cellular biology, neurological diseases, and signaling pathways related to the propofol-induced dysregulated genes.

Supplementary Figure 2 Bioinformatic analysis predicts the molecular networks between propofol-induced dysregulated Ephrin receptor signaling genes and synaptic genes. (**A**) Molecular networks. (**B**) The list of the genes depicted in the Figure A. Genes with red underline are propofol-induced dysregulated Ephrin receptor signaling genes. Genes with green underline are propofol-induced dysregulated synaptic genes. The red and green arrows represent the upregualted and downregulated genes by propofol, respectivelly.
