# Supplementary material for: Integrated Excitatory/Inhibitory Imbalance and Transcriptomic Analysis Reveals the Association between Dysregulated Synaptic Genes and Anesthetic-Induced Cognitive Dysfunction
Source: Cells. 2022 Aug 11;11(16):2497. doi: 10.3390/cells11162497 (PMC9406780; doi:10.3390/cells11162497)
Supplement: Supplementary file 1 [file cells-11-02497-s001.zip › Supplementary Table S1.pdf]

Supplementary Table S1. Propofol-induced dysregulated mRNAs

GeneSymbol	Gene description	P-value	FDR	Fold Change	Regulation
Slc24a5	solute carrier family 24, member 5	0.03	0.37	3.63	up
Gm13139	predicted gene 13139	0.01	0.31	3.35	up
Hk2	hexokinase 2	0.04	0.39	3.17	up
Calm5	calmodulin 5	0.03	0.36	2.62	up
Olfr960	olfactory receptor 960	0.00	0.25	2.54	up
Fam46a	A	0.00	0.25	2.54	up
Arhgef38	Rho guanine nucleotide exchange factor (GEF) 38	0.01	0.28	2.46	up
4921511H03Rik	RIKEN cDNA 4921511H03 gene	0.02	0.35	2.46	up
Hist1h3i	histone cluster 1, H3i	0.01	0.30	2.38	up
Ces1f	carboxylesterase 1F	0.04	0.39	2.31	up
Pdha2	pyruvate dehydrogenase E1 alpha 2	0.03	0.36	2.29	up
Map4	microtubule-associated protein 4	0.01	0.30	2.27	up
Nusap1	nucleolar and spindle associated protein 1	0.01	0.32	2.26	up
Olfr961	olfactory receptor 961	0.01	0.31	2.22	up
Trpm3	transient receptor potential cation channel, subfamily M, member 3	0.05	0.40	2.17	up
Ccdc68	coiled-coil domain containing 68	0.04	0.40	2.17	up
Myo1f	myosin IF	0.04	0.38	2.16	up
Acss3	acyl-CoA synthetase short-chain family member 3	0.00	0.23	2.15	up
Strip2	striatin interacting protein 2	0.00	0.26	2.15	up
Card14	14	0.04	0.38	2.14	up
Cdc6	cell division cycle 6	0.04	0.40	2.14	up
Rgs3	regulator of G-protein signaling 3	0.01	0.30	2.13	up
Lepr	leptin receptor	0.00	0.26	2.11	up
Plekhb1	pleckstrin homology domain containing, family B (evectins) member 1	0.02	0.34	2.10	up
Alb	albumin	0.02	0.35	2.09	up
Ovol2	ovo-like 2 (Drosophila)	0.04	0.39	2.09	up
Ttc37	tetratricopeptide repeat domain 37	0.03	0.36	2.08	up
Dgcr14	DiGeorge syndrome critical region gene 14	0.02	0.36	2.06	up
Chmp4c	charged multivesicular body protein 4C	0.02	0.35	2.05	up
Lepr	leptin receptor	0.03	0.37	2.05	up
Irf6	interferon regulatory factor 6	0.02	0.35	2.04	up
Ccl22	chemokine (C-C motif) ligand 22	0.04	0.39	2.03	up
Olfr984	olfactory receptor 984	0.05	0.40	2.02	up
Zfp750	zinc finger protein 750	0.00	0.09	1.99	up
Gnas	GNAS (guanine nucleotide binding protein, alpha stimulating) complex locus	0.03	0.37	1.98	up
Mta1	metastasis associated 1	0.04	0.40	1.97	up
Flnb	filamin, beta	0.03	0.38	1.95	up
Spint2	serine protease inhibitor, Kunitz type 2	0.02	0.35	1.93	up
Arsg	arylsulfatase G	0.03	0.37	1.93	up
Plekho2	pleckstrin homology domain containing, family O member 2	0.03	0.36	1.92	up
Rccd1	RCC1 domain containing 1	0.01	0.30	1.92	up
Mbnl1	muscleblind-like 1 (Drosophila)	0.02	0.35	1.92	up

Coq5	coenzyme Q5 homolog, methyltransferase (yeast)	0.02	0.35	1.91	up
Olfr507	olfactory receptor 507	0.00	0.27	1.90	up
Trpm3	transient receptor potential cation channel, subfamily M, member 3	0.03	0.36	1.90	up
Spo11	SPO11 meiotic protein covalently bound to DSB homolog (S. cerevisiae)	0.01	0.30	1.87	up
Ccdc114	coiled-coil domain containing 114	0.00	0.26	1.87	up
Gdpd2	glycerophosphodiester phosphodiesterase domain containing 2	0.04	0.39	1.86	up
Prkrip1	Prkr interacting protein 1 (IL11 inducible)	0.01	0.33	1.86	up
Arvcf	armadillo repeat gene deleted in velo-cardio-facial syndrome	0.04	0.40	1.86	up
Nptn	neuroplastin	0.04	0.40	1.86	up
Gmip	Gem-interacting protein	0.04	0.40	1.85	up
Cdkl5	cyclin-dependent kinase-like 5	0.00	0.27	1.84	up
Tnfaip3	protein 3	0.04	0.39	1.84	up
Gm773	predicted gene 773	0.04	0.39	1.84	up
Cd247	CD247 antigen	0.04	0.39	1.84	up
Kcne1	potassium voltage-gated channel, Isk-related subfamily, member 1	0.05	0.40	1.83	up
Ano6	anoctamin 6	0.05	0.40	1.83	up
Sirt6	sirtuin 6	0.01	0.30	1.83	up
Gm5134	predicted gene 5134	0.04	0.38	1.83	up
Myo15	myosin XV	0.04	0.39	1.83	up
Gm13871	predicted gene 13871	0.05	0.40	1.82	up
Tdrd6	tudor domain containing 6	0.02	0.35	1.82	up
Tmem217	transmembrane protein 217	0.03	0.37	1.81	up
Unc5d	unc-5 homolog D (C. elegans)	0.03	0.36	1.81	up
Pappa2	pappalysin 2	0.04	0.40	1.81	up
Myof	myoferlin	0.03	0.36	1.80	up
Myo1c	myosin IC	0.01	0.32	1.80	up
Rxrg	retinoid X receptor gamma	0.04	0.39	1.80	up
Dcun1d1	DCN1, defective in cullin neddylation 1, domain containing 1 (S. cerevisiae)	0.05	0.40	1.80	up
Cdk12	cyclin-dependent kinase 12	0.01	0.31	1.78	up
Frmpd4	FERM and PDZ domain containing 4	0.03	0.36	1.78	up
Cetn4	centrin 4	0.04	0.40	1.77	up
Acbd5	containing 5	0.04	0.40	1.77	up
Ssh2	slingshot homolog 2 (Drosophila)	0.05	0.40	1.76	up
Noxred1	NADP+ dependent oxidoreductase domain containing 1	0.00	0.23	1.75	up
Vmn2r60	vomeronasal 2, receptor 60	0.03	0.36	1.75	up
Mbnl1	muscleblind-like 1 (Drosophila)	0.02	0.35	1.75	up
Pigw	phosphatidylinositol glycan anchor biosynthesis, class W	0.02	0.34	1.75	up
Ccdc129	coiled-coil domain containing 129	0.02	0.36	1.75	up
Vmn2r82	vomeronasal 2, receptor 82	0.03	0.37	1.75	up
Tnfaip8	protein 8	0.04	0.39	1.74	up
Thbs4	thrombospondin 4	0.00	0.25	1.74	up
Krt23	keratin 23	0.04	0.40	1.73	up

Tmem184a	transmembrane protein 184a	0.01	0.33	1.73	up
Pbdc1	polysaccharide biosynthesis domain containing 1	0.02	0.35	1.73	up
Smyd5	SET and MYND domain containing 5	0.00	0.23	1.73	up
Hist1h2bq	histone cluster 1, H2bq	0.00	0.25	1.73	up
Defb25	defensin beta 25	0.04	0.39	1.72	up
Scin	scinderin	0.05	0.40	1.72	up
Aga	aspartylglucosaminidase	0.04	0.40	1.71	up
Top2a	topoisomerase (DNA) II alpha	0.03	0.36	1.71	up
Cops2	COP9 (constitutive photomorphogenic) homolog, subunit 2 (Arabidopsis thaliana)	0.04	0.40	1.71	up
Zfp14	zinc finger protein 14	0.04	0.40	1.71	up
Slc25a25	solute carrier family 25 (mitochondrial carrier, phosphate carrier), member 25	0.04	0.40	1.71	up
Ttc12	tetratricopeptide repeat domain 12	0.03	0.37	1.70	up
Aldh1a3	subfamily A3	0.02	0.35	1.70	up
Fam46c	C	0.05	0.40	1.69	up
Cmya5	cardiomyopathy associated 5	0.02	0.35	1.68	up
Avil	advillin	0.00	0.09	1.68	up
Mdn1	midasin homolog (yeast)	0.05	0.40	1.68	up
Nek11	NIMA (never in mitosis gene a)-related expressed kinase 11	0.02	0.36	1.68	up
Mcidas	multiciliate differentiation and DNA synthesis associated cell cycle protein	0.03	0.37	1.67	up
Itgal	integrin alpha L	0.03	0.38	1.67	up
Rbpj	recombination signal binding protein for immunoglobulin kappa J region	0.00	0.22	1.67	up
Cdk1	cyclin-dependent kinase 1	0.04	0.39	1.66	up
Plekhf1	pleckstrin homology domain containing, family F (with FYVE domain) member 1	0.04	0.39	1.65	up
Lipg	lipase, endothelial	0.03	0.36	1.65	up
Crp	C-reactive protein, pentraxin-related	0.01	0.30	1.65	up
Dab2	phosphoprotein	0.04	0.38	1.64	up
Crh	corticotropin releasing hormone	0.02	0.34	1.64	up
Pask	kinase	0.02	0.35	1.64	up
Khdc3	KH domain containing 3, subcortical maternal complex member	0.01	0.31	1.64	up
Pln	phospholamban	0.03	0.36	1.63	up
Pcsk1	proprotein convertase subtilisin/kexin type 1	0.04	0.38	1.63	up
Zbtb14	zinc finger and BTB domain containing 14	0.04	0.40	1.63	up
Inhba	inhibin beta-A	0.02	0.34	1.62	up
Crip3	cysteine-rich protein 3	0.01	0.33	1.61	up
Olfr481	olfactory receptor 481	0.05	0.40	1.61	up
Gem	GTP binding protein (gene overexpressed in skeletal muscle)	0.00	0.27	1.60	up
Tex19.1	testis expressed gene 19.1	0.05	0.40	1.60	up
Zfp444	zinc finger protein 444	0.03	0.36	1.60	up
Pdlim1	PDZ and LIM domain 1 (elfin)	0.01	0.30	1.60	up
Trip11	thyroid hormone receptor interactor 11	0.01	0.28	1.59	up
Klf10	Kruppel-like factor 10	0.00	0.17	1.59	up
Rrbp1	ribosome binding protein 1	0.04	0.39	1.58	up

Slc28a3	solute carrier family 28 (sodium-coupled nucleoside transporter), member 3	0.02	0.36	1.58	up
Gtf2e2	general transcription factor II E, polypeptide 2 (beta subunit)	0.01	0.31	1.58	up
Pdgfd	polypeptide	0.03	0.37	1.58	up
Alad	aminolevulinate, delta-, dehydratase	0.03	0.36	1.57	up
Rd3	retinal degeneration 3	0.01	0.33	1.57	up
Gulp1	GULP, engulfment adaptor PTB domain containing 1	0.00	0.27	1.57	up
Akap13	A kinase (PRKA) anchor protein 13	0.02	0.36	1.57	up
Klk1b22	kallikrein 1-related peptidase b22	0.04	0.39	1.57	up
Cmip	c-Maf inducing protein	0.03	0.36	1.56	up
Kif1b	kinesin family member 1B	0.00	0.28	1.56	up
Snai1	snail homolog 1 (Drosophila)	0.03	0.37	1.56	up
Smim22	small integral membrane protein 22	0.01	0.29	1.56	up
Elavl2	ELAV (embryonic lethal, abnormal vision, Drosophila)-like 2 (Hu antigen B)	0.04	0.40	1.55	up
Homer1	homer homolog 1 (Drosophila)	0.01	0.31	1.55	up
Eea1	early endosome antigen 1	0.04	0.40	1.55	up
Hsd17b3	hydroxysteroid (17-beta) dehydrogenase 3	0.03	0.37	1.55	up
4933421I07Rik	RIKEN cDNA 4933421I07 gene	0.02	0.36	1.55	up
Odf2	outer dense fiber of sperm tails 2	0.04	0.39	1.55	up
9130008F23Rik	RIKEN cDNA 9130008F23 gene	0.00	0.10	1.54	up
Golim4	golgi integral membrane protein 4	0.04	0.38	1.54	up
Milr1	mast cell immunoglobulin like receptor 1	0.03	0.38	1.54	up
Tmem189	transmembrane protein 189	0.03	0.38	1.54	up
Igtp	interferon gamma induced GTPase	0.04	0.39	1.54	up
Taf7	TAF7 RNA polymerase II, TATA box binding protein (TBP)-associated factor	0.03	0.38	1.53	up
Cdc73	cell division cycle 73, Paf1/RNA polymerase II complex component	0.01	0.28	1.53	up
C2	complement component 2 (within H-2S)	0.01	0.28	1.53	up
P2rx7	purinergic receptor P2X, ligand-gated ion channel, 7	0.01	0.32	1.52	up
Cdca2	cell division cycle associated 2	0.01	0.30	1.52	up
Gpat2	glycerol-3-phosphate acyltransferase 2, mitochondrial	0.05	0.40	1.52	up
BC030867	cDNA sequence BC030867	0.03	0.37	1.51	up
Map3k5	mitogen-activated protein kinase kinase kinase 5	0.02	0.34	1.51	up
Lurap1l	leucine rich adaptor protein 1-like	0.01	0.31	1.51	up
Lamc2	laminin, gamma 2	0.02	0.36	1.51	up
Crbn	cereblon	0.03	0.38	1.51	up
Snapc1	small nuclear RNA activating complex, polypeptide 1	0.04	0.40	1.50	up
Brix1	BRX1, biogenesis of ribosomes, homolog (S. cerevisiae)	0.00	0.09	1.50	up
Edem2	ER degradation enhancer, mannosidase alpha-like 2	0.00	0.10	1.50	up
Filip1	filamin A interacting protein 1	0.00	0.11	3.33	down
Olfr214	olfactory receptor 214	0.01	0.32	2.95	down

Wnt9a	wingless-type MMTV integration site 9A	0.01	0.29	2.58	down
Dbi	diazepam binding inhibitor	0.04	0.39	2.57	down
Styk1	serine/threonine/tyrosine kinase 1	0.03	0.37	2.53	down
Lgr6	leucine-rich repeat-containing G protein-coupled receptor 6	0.02	0.35	2.34	down
Ebf2	early B cell factor 2	0.01	0.30	2.30	down
Serinc2	serine incorporator 2	0.05	0.40	2.26	down
Asz1	ankyrin repeat, SAM and basic leucine zipper domain containing 1	0.02	0.35	2.18	down
Ddt	D-dopachrome tautomerase	0.03	0.37	2.16	down
Sln	sarcolipin	0.01	0.28	2.16	down
Olfr401	olfactory receptor 401	0.03	0.36	2.15	down
Olfr523	olfactory receptor 523	0.01	0.32	2.13	down
Thtpa	thiamine triphosphatase	0.01	0.31	2.11	down
Tdpoz5	TD and POZ domain containing 5	0.01	0.32	2.06	down
Brd8	bromodomain containing 8	0.03	0.36	2.04	down
Eng	endoglin	0.02	0.35	1.96	down
Vmn1r72	vomeronasal 1 receptor 72	0.00	0.09	1.94	down
Lingo4	containing 4	0.00	0.11	1.92	down
Cstad	CSA-conditional, T cell activation-dependent protein	0.00	0.09	1.91	down
Pygo2	pygopus 2	0.01	0.29	1.91	down
Kif16b	kinesin family member 16B	0.01	0.31	1.90	down
Polr3e	polymerase (RNA) III (DNA directed) polypeptide E	0.01	0.29	1.89	down
Dusp13	dual specificity phosphatase 13	0.00	0.25	1.89	down
Slc16a10	solute carrier family 16 (monocarboxylic acid transporters), member 10	0.00	0.23	1.89	down
Dst	dystonin	0.02	0.35	1.88	down
Pan2	PAN2 polyA specific ribonuclease subunit homolog (S. cerevisiae)	0.03	0.37	1.87	down
Cxxc5	CXXC finger 5	0.04	0.40	1.87	down
Pecam1	1	0.00	0.22	1.87	down
Gm5771	predicted gene 5771	0.02	0.34	1.81	down
Gli3	GLI-Kruppel family member GLI3	0.01	0.33	1.81	down
Cybrd1	cytochrome b reductase 1	0.01	0.31	1.80	down
Fam214a	A	0.00	0.19	1.80	down
Ccnk	cyclin K	0.01	0.28	1.79	down
Ndnf	neuron-derived neurotrophic factor	0.01	0.31	1.79	down
Ggnbp1	gametogenetin binding protein 1	0.03	0.36	1.79	down
Ska3	spindle and kinetochore associated complex subunit 3	0.03	0.37	1.79	down
Uqcrh	protein	0.01	0.30	1.78	down
Gpr20	G protein-coupled receptor 20	0.04	0.40	1.77	down
Gab1	growth factor receptor bound protein 2-associated protein 1	0.01	0.29	1.77	down
Cox6a1	polypeptide 1	0.03	0.36	1.76	down
Ace3	angiotensin I converting enzyme (peptidyl-dipeptidase A) 3	0.05	0.40	1.76	down
Camk2b	calcium/calmodulin-dependent protein kinase II, beta	0.02	0.35	1.76	down

2010300C02Rik	RIKEN cDNA 2010300C02 gene	0.01	0.29	1.75	down
Loxhd1	lipoxygenase homology domains 1	0.02	0.35	1.73	down
Tspan32	tetraspanin 32	0.03	0.37	1.73	down
Stox1	storkhead box 1	0.05	0.40	1.72	down
Fbrsl1	fibrosin-like 1	0.03	0.37	1.71	down
Atp9a	ATPase, class II, type 9A	0.04	0.38	1.70	down
Dync1i1	dynein cytoplasmic 1 intermediate chain 1	0.05	0.40	1.70	down
Atp4b	polypeptide	0.00	0.25	1.70	down
Olfr1511	olfactory receptor 1511	0.03	0.36	1.69	down
Smg7	Smg-7 homolog, nonsense mediated mRNA decay factor (C. elegans)	0.02	0.35	1.69	down
Whamm	WAS protein homolog associated with actin, golgi membranes and microtubules	0.01	0.29	1.68	down
Npnt	nephronectin	0.04	0.40	1.67	down
Atp6v0c	ATPase, H+ transporting, lysosomal V0 subunit C	0.02	0.35	1.67	down
Cyp4x1	cytochrome P450, family 4, subfamily x, polypeptide 1	0.00	0.27	1.67	down
Inpp5a	inositol polyphosphate-5-phosphatase A	0.02	0.35	1.67	down
Hsdl1	hydroxysteroid dehydrogenase like 1	0.01	0.31	1.66	down
Tbx1	T-box 1	0.02	0.35	1.66	down
Efna1	ephrin A1	0.01	0.29	1.66	down
Tsga13	testis specific gene A13	0.01	0.33	1.65	down
Tmprss3	transmembrane protease, serine 3	0.04	0.39	1.65	down
Pglyrp3	peptidoglycan recognition protein 3	0.01	0.30	1.65	down
Ecsit	ECSIT homolog (Drosophila)	0.01	0.32	1.65	down
Skap1	src family associated phosphoprotein 1	0.03	0.38	1.65	down
Olfr61	olfactory receptor 61	0.03	0.36	1.64	down
Rtn4	reticulon 4	0.05	0.40	1.64	down
Sctr	secretin receptor	0.02	0.34	1.64	down
Tyk2	tyrosine kinase 2	0.04	0.39	1.64	down
Eras	ES cell-expressed Ras	0.01	0.28	1.63	down
Atp6v1g1	ATPase, H+ transporting, lysosomal V1 subunit G1	0.04	0.40	1.63	down
Tbca	tubulin cofactor A	0.02	0.36	1.63	down
Zcchc5	zinc finger, CCHC domain containing 5	0.03	0.37	1.62	down
Rpl38	ribosomal protein L38	0.04	0.39	1.62	down
Hexim2	hexamethylene bis-acetamide inducible 2	0.01	0.31	1.62	down
Chac1	ChaC, cation transport regulator 1	0.00	0.18	1.61	down
Foxg1	forkhead box G1	0.02	0.35	1.61	down
Mfsd4	containing 4	0.01	0.28	1.61	down
Tusc5	tumor suppressor candidate 5	0.01	0.29	1.61	down
Grtp1	GH regulated TBC protein 1	0.03	0.37	1.61	down
Hyal3	hyaluronoglucosaminidase 3	0.02	0.35	1.60	down
Nsmf	NMDA receptor synaptonuclear signaling and neuronal migration factor	0.03	0.36	1.60	down
Mmrn2	multimerin 2	0.01	0.31	1.60	down
Higd2a	HIG1 domain family, member 2A	0.04	0.39	1.60	down
Wnt9b	wingless-type MMTV integration site 9B	0.05	0.40	1.60	down
Sppl3	signal peptide peptidase 3	0.01	0.32	1.60	down
Sptbn4	spectrin beta, non-erythrocytic 4	0.03	0.37	1.60	down

Grina	glutamate receptor, ionotropic, N-methyl D-aspartate-associated protein 1 (glutamate binding)	0.04	0.39	1.60	down
Caml	calcium modulating ligand	0.04	0.39	1.60	down
Sfta2	surfactant associated 2	0.00	0.25	1.59	down
Tcf12	transcription factor 12	0.04	0.40	1.58	down
Prl2c5	prolactin family 2, subfamily c, member 5	0.03	0.36	1.58	down
Pctp	phosphatidylcholine transfer protein	0.03	0.37	1.58	down
Cpn2	carboxypeptidase N, polypeptide 2	0.01	0.31	1.57	down
Mcrs1	microspherule protein 1	0.01	0.31	1.57	down
Ptges2	prostaglandin E synthase 2	0.05	0.40	1.57	down
Rbm14	RNA binding motif protein 14	0.01	0.32	1.57	down
Ndrg4	N-myc downstream regulated gene 4	0.03	0.37	1.57	down
Tcf12	transcription factor 12	0.02	0.36	1.57	down
Krtap4-16	keratin associated protein 4-16	0.02	0.35	1.56	down
Nfib	nuclear factor I/B	0.00	0.22	1.56	down
Htr6	5-hydroxytryptamine (serotonin) receptor 6	0.05	0.40	1.55	down
Oxct2b	3-oxoacid CoA transferase 2B	0.02	0.33	1.55	down
Rps27a	ribosomal protein S27A	0.04	0.40	1.55	down
Eme1	essential meiotic endonuclease 1 homolog 1 (S. pombe)	0.03	0.36	1.55	down
Cldn5	claudin 5	0.03	0.36	1.55	down
Defb29	defensin beta 29	0.05	0.40	1.55	down
Pcdhgc5	protocadherin gamma subfamily C, 5	0.04	0.39	1.55	down
Adrbk1	adrenergic receptor kinase, beta 1	0.00	0.27	1.55	down
Rabggtb	RAB geranylgeranyl transferase, b subunit	0.02	0.35	1.55	down
Atp13a1	ATPase type 13A1	0.03	0.37	1.54	down
Nolc1	1	0.04	0.40	1.54	down
Npcd	neuronal pentraxin chromo domain	0.00	0.26	1.54	down
Mrps2	mitochondrial ribosomal protein S2	0.05	0.40	1.54	down
Ptprb	type, B	0.01	0.32	1.54	down
Pnpla3	containing 3	0.00	0.23	1.54	down
A330050F15Rik	RIKEN cDNA A330050F15 gene	0.00	0.23	1.54	down
Ttc17	tetratricopeptide repeat domain 17	0.02	0.35	1.54	down
Cxcl12	chemokine (C-X-C motif) ligand 12	0.02	0.36	1.54	down
Cxcr5	chemokine (C-X-C motif) receptor 5	0.03	0.37	1.54	down
Sec61g	SEC61, gamma subunit	0.00	0.25	1.53	down
Maml3	mastermind like 3 (Drosophila)	0.02	0.35	1.53	down
Wdr33	WD repeat domain 33	0.04	0.38	1.53	down
Ccdc101	coiled-coil domain containing 101	0.00	0.15	1.53	down
Mapk1ip1	mitogen-activated protein kinase 1 interacting protein 1	0.01	0.30	1.53	down
Zdhhc7	zinc finger, DHHC domain containing 7	0.02	0.34	1.53	down
Thbs1	thrombospondin 1	0.00	0.23	1.53	down
Lsm2	LSM2 homolog, U6 small nuclear RNA associated (S. cerevisiae)	0.01	0.28	1.53	down
Slc1a2	solute carrier family 1 (glial high affinity glutamate transporter), member 2	0.04	0.39	1.53	down
Clspn	claspin	0.01	0.29	1.53	down
Atf3	activating transcription factor 3	0.03	0.38	1.53	down
Lcn4	lipocalin 4	0.02	0.35	1.53	down

Cryab	crystallin, alpha B	0.02	0.35	1.52	down
Rapgef3	Rap guanine nucleotide exchange factor (GEF) 3	0.03	0.37	1.52	down
Rab37	RAB37, member of RAS oncogene family	0.02	0.36	1.52	down
5031439G07Rik	RIKEN cDNA 5031439G07 gene	0.00	0.27	1.52	down
Actn3	actinin alpha 3	0.00	0.23	1.52	down
Ctbp1	C-terminal binding protein 1	0.03	0.37	1.52	down
Prom1	prominin 1	0.01	0.30	1.52	down
Myh11	[illegible] muscle	0.04	0.39	1.51	down
Spock1	sparc/osteonectin, cwcv and kazal-like domains proteoglycan 1	0.01	0.28	1.51	down
Apol10b	apolipoprotein L 10B	0.00	0.25	1.51	down
Ephx2	epoxide hydrolase 2, cytoplasmic	0.04	0.39	1.51	down
Phf12	PHD finger protein 12	0.03	0.38	1.51	down
Lgmn	legumain	0.01	0.28	1.51	down
Kcnma1	[illegible] activated channel, subfamily M, alpha member 1	0.05	0.40	1.51	down
Scn4b	sodium channel, type IV, beta	0.04	0.40	1.51	down
Cdc25b	cell division cycle 25B	0.03	0.37	1.51	down
4833439L19Rik	RIKEN cDNA 4833439L19 gene	0.01	0.33	1.51	down
Plekhg5	pleckstrin homology domain containing, family G (with RhoGef domain) member 5	0.00	0.17	1.50	down
Ube2h	ubiquitin-conjugating enzyme E2H	0.02	0.35	1.50	down
Tnik	TRAF2 and NCK interacting kinase	0.05	0.40	1.50	down
Rabggtb	RAB geranylgeranyl transferase, b subunit	0.04	0.38	1.50	down

Note: false discovery rate (FDR)
