# Supplementary material for: Integrated Excitatory/Inhibitory Imbalance and Transcriptomic Analysis Reveals the Association between Dysregulated Synaptic Genes and Anesthetic-Induced Cognitive Dysfunction
Source: Cells. 2022 Aug 11;11(16):2497. doi: 10.3390/cells11162497 (PMC9406780; doi:10.3390/cells11162497)
Supplement: Supplementary file 1 [file cells-11-02497-s001.zip › Supplementary Table S2.pdf]

Supplementary Table S2. Propofol-induced dysregulated synapse genes involved in nervous system development and function

Genes	Diseases or Functions Annotation
CAMK2B,NSMF	Object recognition memory
NSMF,RTN4,TNIK	Formation of dendrites
RTN4	Functional recovery of axons
KCNMA1	Eyelid reflex
RTN4	Projection of motor axons
RTN4	Sprouting of corticospinal neurons
RTN4	Projection of sensory axons
CAMK2B,SLC1A2,TNIK	Cell viability of neurons
CAMK2B,NSMF,RTN4	Long-term potentiation
RTN4	Inhibition of dorsal root ganglion cells
RTN4	Regeneration of pyramidal tract
RTN4	Branching of peripheral nerve
CAMK2B,NSMF,RTN4,TNIK	Neuritogenesis
RTN4,TNIK	Regeneration of axons
RTN4	Binding of neurites
KCNMA1,RPL38,RTN4	Sensation
NSMF,RTN4	Outgrowth of axons
KCNMA1,NSMF	Innervation of tissue
CAMK2B,NSMF,RTN4,SLC1A2	Morphology of nervous tissue
NSMF,SLC1A2	Abnormal morphology of hippocampus
RTN4	Growth of raphespinal axon
RTN4	Growth of corticospinal axon
CAMK2B,NSMF,RTN4	Outgrowth of neurites
KCNMA1	Blinking
KCNMA1,RTN4,SLC1A2	Differentiation of neurons
CAMK2B,NSMF	Plasticity of synapse
RTN4	Myelination of optic nerve
RTN4	Proliferation of cerebellar granule cell
KCNMA1,NSMF	Action potential of neurons
KCNMA1,NSMF,SLC1A2	Neurotransmission
SLC1A2	Loss of hippocampal neurons
SLC1A2	Formation of cerebral neocortex
KCNMA1	Function of inner hair cells
CAMK2B	Survival of cerebellar granule cell
KCNMA1,RPL38	Hearing
RTN4	Outgrowth of dorsal root ganglion cells
KCNMA1	Innervation of inner hair cells
RTN4	Outgrowth of brain cells
RTN4	Size of dendritic trees
RTN4	Proliferation of cortical neurons
SLC1A2,TNIK	Development of cerebral cortex
NSMF,SLC1A2	Abnormal morphology of telencephalon
SLC1A2	Abnormal morphology of hippocampal CA1 region
RTN4	Size of growth cone
RTN4	Remyelination of axons

CAMK2B,RTN4	Myelination
CAMK2B,KCNMA1	Coordination
CAMK2B	Thickness of myelin sheath
RTN4	Migration of neuroblasts
TNIK	Survival of retinal ganglion cells
CAMK2B	Long-term potentiation of collateral synapses
NSMF	Plasticity of neuronal synapse
RTN4,SLC1A2	Migration of neurons
NSMF,RTN4,SLC1A2	Morphology of neurons
KCNMA1	Differentiation of hair cells
TNIK	Neurogenesis of dentate gyrus
RTN4,SLC1A2,TNIK	Development of central nervous system
NSMF	Abnormal morphology of striatum
RTN4	Fasciculation of axons
SLC1A2	Cell viability of motor neurons
CAMK2B,RTN4	Branching of neurites
KCNMA1,SLC1A2	Synaptic transmission
RTN4	Formation of dendritic spines
NSMF	Excitatory postsynaptic potential of neurons
CAMK2B	Long term synaptic depression of synapse
RTN4	Growth of dendrites
