# Supplementary material for: Integrated Excitatory/Inhibitory Imbalance and Transcriptomic Analysis Reveals the Association between Dysregulated Synaptic Genes and Anesthetic-Induced Cognitive Dysfunction
Source: Cells. 2022 Aug 11;11(16):2497. doi: 10.3390/cells11162497 (PMC9406780; doi:10.3390/cells11162497)
Supplement: Supplementary file 1 [file cells-11-02497-s001.zip › Supplementary Table S3.pdf]

Supplementary Table S3. Propofol-induced downregulated synapse genes related to neurological disorders

Genes	Diseases or Functions Annotation
CAMK2B,CTBP1,KCNMA1,SLC1A2	Ataxia
CAMK2B,CTBP1,KCNMA1,NSMF,SLC1A2,TNIK	Cognitive impairment
CAMK2B,CTBP1,KCNMA1,NSMF,PLEKHG5,RTN4,SLC1A2,TNIK	Familial neurological disorder
CAMK2B,CTBP1,KCNMA1,SLC1A2,TNIK	Mental retardation
CAMK2B,KCNMA1,NSMF,PLEKHG5,RTN4,SLC1A2,TNIK	Familial encephalopathy
CAMK2B,KCNMA1,PLEKHG5,RTN4,SLC1A2,TNIK	Progressive neurological disorder
CTBP1,KCNMA1,SLC1A2	Familial ataxia
NSMF,RTN4,SLC1A2	Sensory disorders
KCNMA1,PLEKHG5,SLC1A2	Spinocerebellar disease
KCNMA1,PLEKHG5,RTN4,SLC1A2	Degenerative brain disorder
NSMF	Susceptibility to Kallmann syndrome type 9
KCNMA1	Liang-Wang syndrome
TNIK	Autosomal recessive mental retardation type 54
CTBP1	Hypotonia, ataxia, developmental delay, and tooth enamel defect syndrome
CAMK2B	Autosomal dominant mental retardation type 54
KCNMA1	Cerebellar atrophy, developmental delay and seizures
NSMF	Susceptibility to hypogonadotropic hypogonadism 9 without anosmia
RTN4	Acute cerebral infarction
PLEKHG5	Charcot-Marie-Tooth disease recessive intermediate type C
SLC1A2	Early infantile epileptic encephalopathy type 41
PLEKHG5	Facioscapulohumeral spinal muscular atrophy

KCNMA1,SLC1A2	Spinocerebellar ataxia type 7
PLEKHG5,SLC1A2	Familial amyotrophic lateral sclerosis
CAMK2B,PLEKHG5,RTN4,SLC1A2,TNIK	Progressive encephalopathy
PLEKHG5	Autosomal recessive distal spinal muscular atrophy type 4
KCNMA1	Paroxysmal nonkinesigenic dyskinesia type 3
RTN4	Focal cortical dysplasia of Taylor type IIB
SLC1A2	Damage of CA1 neuron
KCNMA1,PLEKHG5,SLC1A2	Hereditary neuropathy
CAMK2B,KCNMA1,SLC1A2	Autosomal dominant encephalopathy
KCNMA1,PLEKHG5,RTN4,SLC1A2	Progressive motor neuropathy
CAMK2B,CTBP1,KCNMA1	Nonspecific mental retardation
SLC1A2	Progressive stroke
NSMF,SLC1A2	Abnormal morphology of hippocampus
KCNMA1,SLC1A2	Familial generalized epilepsy
PLEKHG5,RTN4,SLC1A2	Amyotrophic lateral sclerosis
CAMK2B,CTBP1,KCNMA1,RTN4,SLC1A2	Movement Disorders
SLC1A2	Delay in amyotrophic lateral sclerosis
CAMK2B,SLC1A2,TNIK	Familial mental retardation
RTN4,SLC1A2	Brain developmental abnormality
SLC1A2	Loss of hippocampal neurons
KCNMA1,PLEKHG5	Hereditary motor and sensory neuropathy
RTN4,SLC1A2	Hyperesthesia
PLEKHG5	Juvenile amyotrophic lateral sclerosis
CAMK2B,KCNMA1,RTN4,SLC1A2	Disorder of basal ganglia
RTN4,SLC1A2,TNIK	Schizophrenia
SLC1A2	Cell death of CA1 neuron
KCNMA1	Hereditary spastic ataxia
SLC1A2	Neurodegeneration of pyramidal neurons
KCNMA1	Noise-induced hearing loss
NSMF,SLC1A2	Abnormal morphology of telencephalon
SLC1A2	Thermal hypalgesia

SLC1A2	Abnormal morphology of hippocampal CA1 region
SLC1A2	Cerebral edema
PLEKHG5,SLC1A2	Pediatric-onset neurological disease
RTN4	Tuberous sclerosis complex
SLC1A2	Cerebral amyloid angiopathy
KCNMA1	Bronchospasm
PLEKHG5,SLC1A2	Paralysis
NSMF,SLC1A2	Major depression
SLC1A2	Spinocerebellar ataxia type 2
CAMK2B,KCNMA1,RTN4,SLC1A2	Neuromuscular disease
CAMK2B,SLC1A2,TNIK	Alzheimer disease
NSMF,RTN4,SLC1A2	Congenital encephalopathy
RTN4	Heat hyperalgesia
SLC1A2	Cervical spondylotic myelopathy
KCNMA1,SLC1A2	Degeneration of neurons
RTN4,SLC1A2	Stroke
KCNMA1,RTN4	Multiple Sclerosis
KCNMA1	Short stride length
NSMF	Abnormal morphology of striatum
SLC1A2	Multiple system atrophy
CAMK2B,KCNMA1	Pervasive developmental disorder
CAMK2B,KCNMA1,SLC1A2	Dyskinesia
KCNMA1,RTN4,SLC1A2	Epilepsy or neurodevelopmental disorder
KCNMA1	Neurodegeneration of outer hair cells
SLC1A2	Fragile X syndrome
SLC1A2	Progressive supranuclear palsy
KCNMA1	Idiopathic generalized epilepsy
RTN4,SLC1A2	Familial neurodevelopmental disorder
SLC1A2	Tourette syndrome
SLC1A2	Allodynia
