# Supplementary material for: Integrated Excitatory/Inhibitory Imbalance and Transcriptomic Analysis Reveals the Association between Dysregulated Synaptic Genes and Anesthetic-Induced Cognitive Dysfunction
Source: Cells. 2022 Aug 11;11(16):2497. doi: 10.3390/cells11162497 (PMC9406780; doi:10.3390/cells11162497)
Supplement: Supplementary file 1 [file cells-11-02497-s001.zip › Supplementary Table S4.pdf]

Supplementary Table S4. Sequence information for primers

Gene name	Sequence (from 5' to 3')	PCR Product length (bp)
Filip1	F: GCTGGAAAACGAGAAGCACAAGC<br>R: TTGAGCCGCTTGGCGTTTTCCT	156
Nsmf	F: TTCTTGCCACCCAAGGTCATGC<br>R: TCCAGGATGTCCTCGAAGGTTG	140
Actin	F: AAGAGCTATGAGCTGCCTGA<br>R: TACGGATGTCAACGTCACAC	160

Note: F: forward primer; R: reverse primer. The full gene names of the propofol-induced dysregulated mRNAs are detailed in Supplementary Table 1.
